# Anchorage of bacterial effector at plasma membrane *via* selective phosphatidic acid binding to modulate host cell signaling

**DOI:** 10.1371/journal.ppat.1012694

**Published:** 2024-11-12

**Authors:** Meng Wang, Qixiao Guan, Chunyan Wang, Lyubin Hu, Xueyan Hu, Menglin Xu, Yuhao Cai, Haoran Zhang, Qing Cao, Huiming Sheng, Xiaohui Wei, Jane E. Koehler, Hongjing Dou, Ruo-xu Gu, Congli Yuan

**Affiliations:** 1 Shanghai Key Laboratory of Veterinary Biotechnology, School of Agriculture and Biology, Shanghai Jiao Tong University, Shanghai, China; 2 School of materials Science and Engineering, Shanghai Jiao Tong University, Shanghai, China; 3 School of Life Sciences and Biotechnology, Shanghai Jiao Tong University, Shanghai, China; 4 Shanghai Children’s Medical Center, Shanghai Jiao Tong University, Shanghai, China; 5 Tongren Hospital, Shanghai Jiao Tong University, Shanghai, China; 6 School of Pharmaceutical Sciences, Shanghai Jiao Tong University, Shanghai, China; 7 Department of Medicine, Division of Infectious Diseases, and the Microbial Pathogenesis and Host Defense Program, University of California, San Francisco, California, United States of America; Institut Pasteur, FRANCE

## Abstract

Binding phospholipid is a simple, yet flexible, strategy for anchorage of bacterial effectors at cell membrane to manipulate host signaling responses. Phosphatidylinositol 4-phosphate and phosphatidylinositol 4,5-biphosphate are the only two phospholipid species known to direct bacterial effectors to establish inner leaflet localization at the plasma membrane. Here, selectivity of phosphatidic acid (PA) by bacterial effectors for the plasma membrane anchorage and its molecular entity was identified. C-terminal BID domain of *Bartonella* T4SS effectors (Beps) directed the plasma membrane localization of Beps in host cells through binding with PA. A hydrophobic segment of the ‘HOOK’ subdomain from BID is inserted into the bilayer to enhance the interaction of positively charged residues with the lipid headgroups. Mutations of a conserved arginine facilitating the electrostatic interaction, a conserved glycine maintaining the stability of the PA binding groove, and hydrophobic residues determining membrane insertion, prevented the anchorage of Beps at the plasma membrane. Disassociation from plasma membrane to cytosol attenuated the BepC capacity to induce stress fiber formation and cell fragmentation in host cells. The substitution of alanine with aspartic acid at the -1 position preceding the conserved arginine residue hindered BepD anchoring at the plasma membrane, a vital prerequisite for its ability to elicit IL-10 secretion in host macrophages. In conclusion, our findings reveal the PA-binding properties of bacterial effectors to establish plasma membrane localization and will shed light on the intricate mechanisms employed by bacterial effectors within host cells.

## Author summary

Understanding the plasma membrane localization of bacterial effectors is crucial to illuminate their mechanism of regulation of host cell signaling. PtdIns(4,5)P_2_ is a labeling phospholipid of the plasma membrane that bacterial effectors usually select to anchor them at the plasma membrane. Here, we described *Bartonella* effector proteins (Beps) bind to phosphatidic acid to establish a localization at the inner leaflet of plasma membrane *via* a conserved BID domain. By the combined results from computational simulations and experimental measurements, we elucidated specific properties underlying the PA-binding of *Bartonella* Beps. We also demonstrated the crucial role of plasma membrane anchorage through PA-binding in regulating cytoskeletal rearrangement and immune response by Beps.

## Introduction

The virulence of various bacterial pathogens relies on their ability to secrete effector proteins into host cells *via* specialized secretion systems. These effector proteins reprogram host signaling responses, involving immune detection, cytoskeleton rearrangement, and vesicular trafficking, through specific subcellular localization [1,2]. The host cell membrane serves as a critical site for the accumulation of bacterial effector proteins, acting as a hub for regulating host cell signals. Approximately 30% of total bacterial effectors are associated with the plasma membrane and other membrane-containing organelles in eukaryotic cells, achieved through lipidation, transmembrane domains, and lipid binding [3]. For example, the *Salmonella* effector SifA undergoes isoprenoid modification and S-acylation within host cells, allowing its localization at the plasma membrane and inducing the formation of *Salmonella*-induced tubules for bacterial intracellular survival and proliferation [4]. Similarly, SteD maintains a transmembrane domain, facilitating its integration into the membranes of the endoplasmic reticulum (ER)/Golgi network, where it hijacks the host AP1-mediated trafficking pathway to reach major histocompatibility complex II (MHCII) compartments [5]. Additionally, the *Legionella* effector SidC targets the *Legionella*-containing vacuole (LCV) membrane by binding to phosphatidylinositol 4-phosphate (PtdIns4P), promoting the recruitment of the ER to the LCV [6]. Among these mechanisms, binding to phospholipids emerges as the most efficient strategy for anchoring bacterial effectors to the cell membrane. Unlike many conserved domains identified in eukaryotic proteins that bind to phospholipids, consensus sequences or motifs are scarcely identified in bacterial effectors.

*Bartonella* species are facultative intracellular pathogens with remarkable adaptations to their specific mammalian hosts and vector reservoirs [7]. *Bartonella* employs its VirB/VirD4 type IV secretion system (T4SS) to translocate a cocktail of evolutionarily related *Bartonella* effector proteins (Beps) into host cells [8]. Beps are multi-domain proteins primarily featuring an N-terminal filamentation induced by cAMP (FIC) domain or tandemly-repeated tyrosine motifs phosphorylated by host kinases, a C-terminal *Bartonella* intracellular delivery (BID) domain acting as a bipartite signal for T4SS-mediated translocation, and a positively charged C-terminus [9,10]. Previous studies have reported the plasma membrane localization of some Beps [11–15]. For instance, BepA associates with the plasma membrane-bound enzyme adenylyl cyclase isoform 7 to increase intracellular cAMP levels and prevent host cell apoptosis [11,16]. BepC’s plasma membrane localization induces stress fiber formation by facilitating the anchorage of GEF-H1 to the plasma membrane from microtubules [12,13]. Plasma membrane anchorage of BepG facilitates a novel intracellular infection mode of *Bartonella* called "invasome" (engulfment of large bacterial aggregates) [14]. However, the molecular entity responsible for the plasma membrane localization of Beps and the potential mechanism of regulated binding remain elusive. In this study, we identified a conserved phosphatidic acid (PA) binding motif (PABM) in the BID domain enabling Beps to precisely localize to the plasma membrane. Plasma membrane localization is pivotal for the reported biological functions of Beps. Moreover, this highly specific PABM is also expected to serve as an effective biosensor for studying PA dynamics at the plasma membrane.

## Results

### Anchorage of *Bartonella* effectors at the plasma membrane is determined by the BID domain

**Fig 1:**
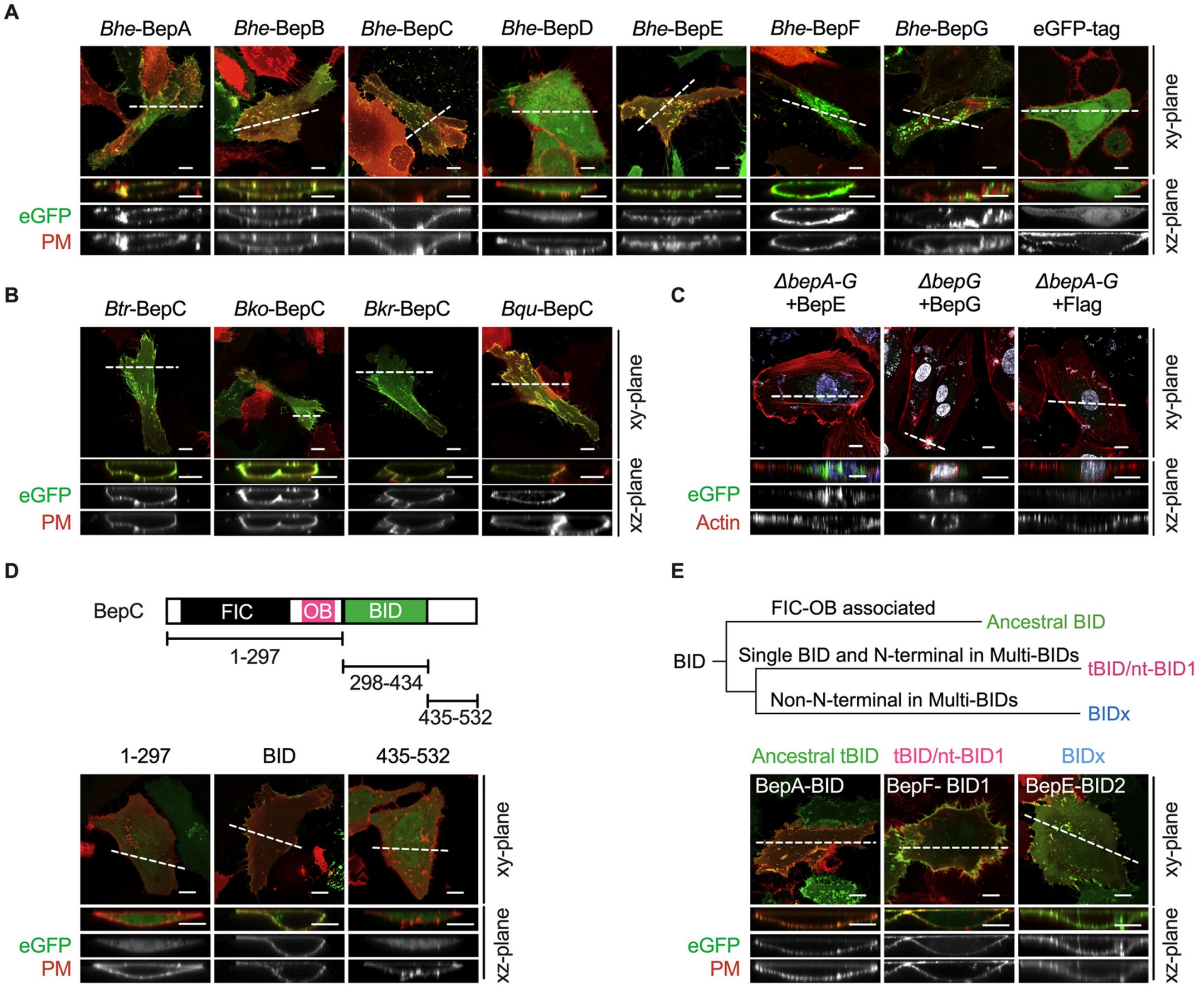
BID domain contributes the plasma membrane localization of *Bartonella* effectors. (A) Ectopic expression of *Bhe*-BepA to BepG fused with eGFP tag was investigated in HeLa cells, followed by fixation, staining with membrane probe (DiD red) and confocal microscope investigation. (B) BepC orthologues from *Btr*, *Bqu*, *Bko*, *Bkr* strains were investigated in HeLa cells, followed by fixation, staining with membrane probe (DiD red) and confocal microscopy. (C) HUVECs were infected for 24 hours with a multiplicity of infection (MOI) = 300 of the effector-deficient mutant strain with isogenic expression of *bepE* (Δ*bepA-G*+*bepE*), the *bepG* deleted *Bhe* strain complemented with IPTG-induced *bepG* (Δ*bepG*+*bepG*) or a control strain (*Bhe*-Δ*bepG*+*Flag*-tag). Cells were fixed, stained and localization of T4SS translocated BepE and BepG was analyzed by confocal microscopy. F-actin is represented in red (phalloidin), bacteria and the cell nucleus in white (DAPI), and BepE-Flag and BepG-Flag in green. (D) BepC truncations fused with eGFP tag either containing FIC-OB (1–297), BID (298–434) and C-terminus (435–532) were transfected in HeLa cells, plasma membrane localization was visualized by DiD staining. (E) Classification scheme of BID domains (above). Phylogenetic tree with species names and UniProt IDs can be found in S1 Fig. Localization of three representatives of ancestral tBID (BepA-BID), ntBID1 (BepF-BID1) and BIDx (BepE-BID2) were visualized by DiD staining. All experiments were performed more than three times independently, and representative images acquired in xy-plane and xz-plane. Scale bars = 10 μm.

To explore the subcellular distribution of *Bartonella* effector proteins (Beps) within host cells, we transiently expressed BepA to BepG from *Bartonella henselae* (*Bhe*), each fused with an eGFP tag, in HeLa cells. Notably, while most Beps anchored at the plasma membrane, BepD uniquely displayed cytosolic localization (Fig 1A). To ascertain whether plasma membrane localization of *Bartonella* T4SS effectors is consistent across various *Bartonella* species, we ectopically expressed BepC from *B*. *tribocorum* (*Btr*), *B*. *kosoyi* (*Bko*), *B*. *krasnovii* (*Bkr*), or *B*. *quintana* (*Bqu*) in HeLa cells. As expected, all BepC proteins localized to the plasma membrane and induced cell fragmentation (Fig 1B). Subsequently, we examined the subcellular localization of naturally translocated Beps by infecting endothelial cells for 24 hours. We used a Bep locus-deleted *Bhe* mutant strain (Δ*bepA-G*) carrying a plasmid encoding BepE-Flag or Flag-tag (invasion through single bacilli endocytosis), or a *bepG*-deleted *Bhe* strain complemented with *bepG* (invasion through invasome). Immunofluorescent labeling revealed that T4SS-translocated BepE and BepG also localized to the plasma membrane (Fig 1C). Additionally, when *Bartonella* aggregates were engulfed by endothelial cells, BepG was found to be enriched in the surrounding area of the invasome structure (Fig 1C).

Beps possess either a FIC-OB fold or tandem-repeat tyrosine-phosphorylation motifs at their N-terminus and one or multiple conserved BID domains at their C-terminus, reported as substrates for recognition by T4SS translocation [17,18]. Given the universality of subcellular localization and the conservation of structural domains in Beps, it is hypothesized that the C-terminal region containing the BID domain determines their localization at the plasma membrane [12,13]. Therefore, we generated truncated variants containing the N-terminal FIC-OB fold (1–297), BID domain (298–434), or C-terminal positively charged tail (435–532) of *Bhe* BepC (Fig 1D). Confocal microscopy revealed that the BID domain, rather than the N-terminal FIC-OB fold or C-terminal tail, localized to the plasma membrane (Fig 1D). Phylogenetically, the BID domains of Beps in *Bartonella spp*. are categorized into three groups: FIC-OB-combined BID (defined as ancestral tBID), BID from single BID effector and N-terminal one from multiple BID effectors (tBID/nt-BID1), non-N-terminal BIDs from multiple BID effectors (BIDx), where "x" denotes the sequential order of the BID domain from the N-terminus (Figs 1E and S1). One representative from each of the three BID groups, BepA-BID (ancestral tBID), N-terminal BepF-BID (nt-BID1), and BepE-BID2 (BIDx), was transiently expressed in HeLa cells. Results confirmed the anchorage of all tested representatives at the plasma membrane (Fig 1E).

### *Bartonella* effectors localize at the plasma membrane by binding to anionic phospholipids

**Fig 2:**
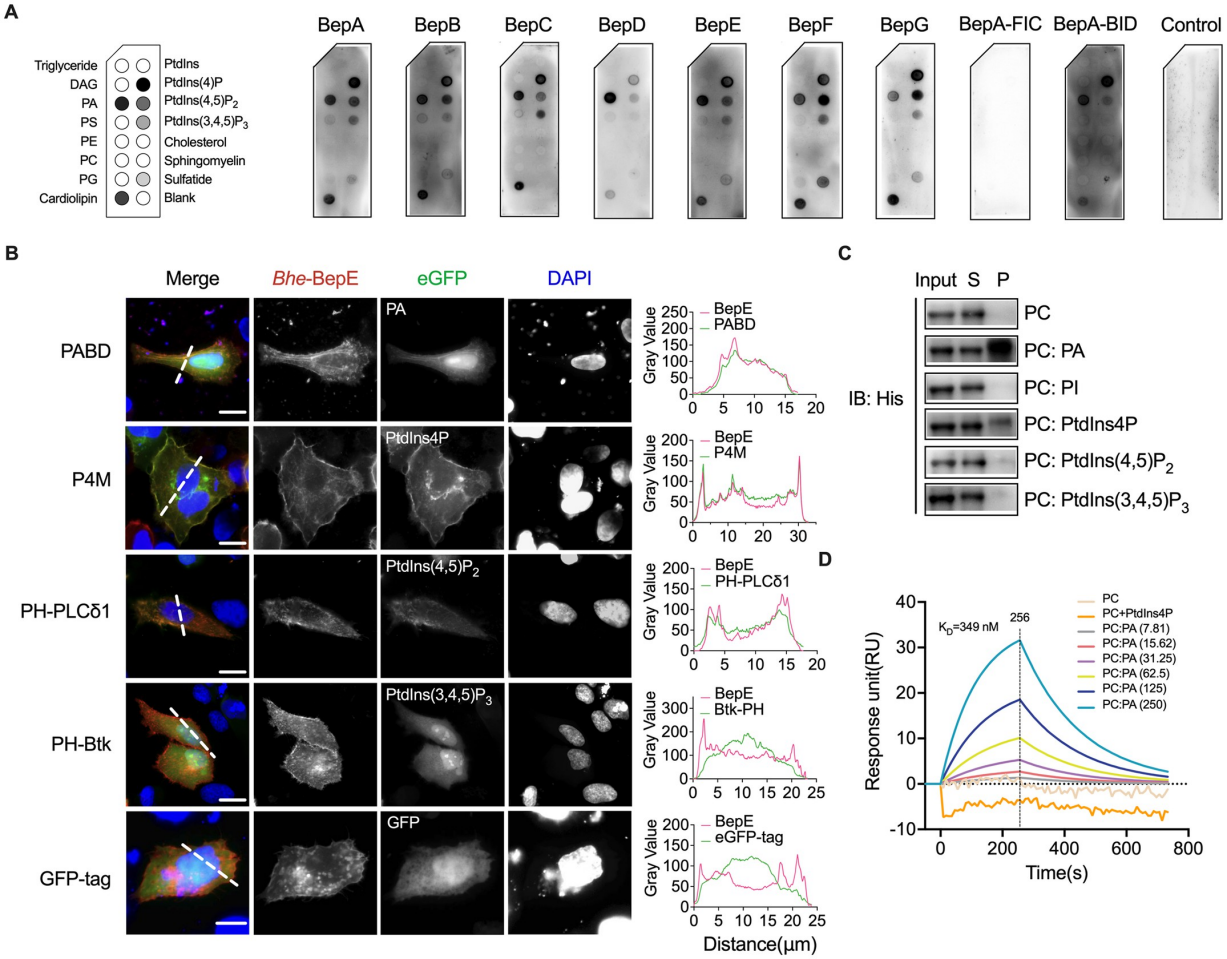
The BID domain interacts with negatively charged phospholipids. (A) Purified *Bhe*-BepA to BepG, BepA-FIC or BepA-BID fused with Flag tag were overlaid on membrane lipid strips. 3x Flag tag was used as control. DAG, diacylglycerol; PA, phosphatidic acid; PS, phosphatidylserine; PE, phosphatidylethanolamine; PC, phosphatidylcholine; PG, phosphatidylglycerol. (B) HeLa cells were co-transfected with plasmids expressing *Bhe bepE* with mRFP and lipid biosensors with eGFP tag. Cells were then fixed and analyzed by fluorescence microscopy. DNA in blue (DAPI). Fluorescence intensity was plotted along the lines. PA, PtdIns4P, PtdIns(4,5)P_2_ and PtdIns(3,4,5)P_3_ pools at the plasma membrane are labeled by PABD (Spo20p 51–91) [54], P4M (*L*. *pneumophila* SidM 546–647) [55], PH-PLCδ1 [56] and PH-Btk [56], respectively. (C) His-tagged BepE-BID1 was incubated with liposomes containing 100% PC, 90% PC and 10% PI, 90% PC and 10% PA, 90% PC and 10% PtdIns4P, 90% PC and 10% PtdIns(4,5)P_2_ or 90% PC and 10% PtdIns(3,4,5)P_3_. Following liposomes ultracentrifugation, anti-His antibodies were used to detect BepE-BID1 in the pellet (P; bound to liposomes) or the supernatant (S; unbound to liposomes) fraction of samples. (D) SPR analysis showing PC (250 μM), PC: PA (0 to 250 μM) or PC: PtdIns4P (250 μM) liposomes binding to immobilized BepE-BID1 on LifeDisc biosensors (Highly Sensitive) chip. The K_D_ values were calculated by using the WeSPR 200 software. All experiments were performed in three independent experiments, and representative data are shown. Scale bars = 10 μm.

Due to the absence of potential lipidation sites and the lack of hydrophobic transmembrane regions in Beps, we excluded the possibility of Beps localizing to the cell membrane through lipidation and transmembrane domains. Therefore, we focused on examining the membrane localization of Beps through lipid binding. We conducted protein lipid overlay assays, where various membrane lipids were spotted onto the strips and incubated with all Beps from the *Bhe* strains. Our data revealed that Beps selectively interact with anionic phospholipids, with strongest binding to phosphatidic acid (PA), cardiolipin and phosphatidylinositol 4-phosphate (PtdIns4P), weaker binding to phosphatidylinositol 4,5-bisphosphate [PtdIns(4,5)P_2_], and phosphatidylinositol 3,4,5-trisphosphate [PtdIns(3,4,5)P_3_] (Fig 2A). The BID domains were observed to bind with lipid species in a manner consistent with full-length Beps, while the FIC domain did not show any binding to lipid species (Fig 2A). Cardiolipin, predominantly localized in the inner mitochondrial membranes, was excluded from further studies.

Given that BepE is the first *Bartonella* effector whose BID1 crystal structure has been resolved (PDB ID: 4YK3), BepE was selected for the following studies [18]. We co-transfected BepE and biosensors specific to the aforementioned phospholipids into HeLa cells. Fluorescence microscopy analysis demonstrated that BepE co-localized with biosensors of PA (Spo20P-51-91), PtdIns4P (P4M), and PtdIns(4,5)P_2_ (PH-PLCδ1), but not PtdIns(3,4,5)P_3_ (PH-Btk) (Fig 2B). To further investigate the interaction between Beps and a genuine lipid bilayer, liposomes consisting of 90% phosphatidylcholine (PC) and 10% of the interested phospholipids were utilized. Dynamic light scattering (DLS) measurements confirmed the uniform size distribution of the liposomes, and cryo-transmission electron microscopy (Cryo-TEM) observation revealed their spherical morphology (S2 Fig). Purified BepE-BID1 with His-tag was incubated with the target liposomes, followed by ultracentrifugation. Our results showed the strongest association of BepE-BID1 with the PC: POPA liposomes, followed by the PC: PtdIns4P liposomes. Binding to liposomes containing PtdIns(4,5)P_2_ or PtdIns(3,4,5)P_3_ was barely observed (Fig 2C). The Surface Plasmon Resonance (SPR) analysis provided a quantitative assessment of the interaction between BepE-BID1 and liposomes, specifically highlighting a notable binding affinity towards POPA-containing liposomes with an estimated K_D_ of 349 nM (Fig 2D). No significant interaction with PC or PC: PtdIns4P liposomes was identified (Fig 2D).

### Identification of the BID-lipid interaction and critical amino acids determining plasma membrane anchorage

**Fig 3:**
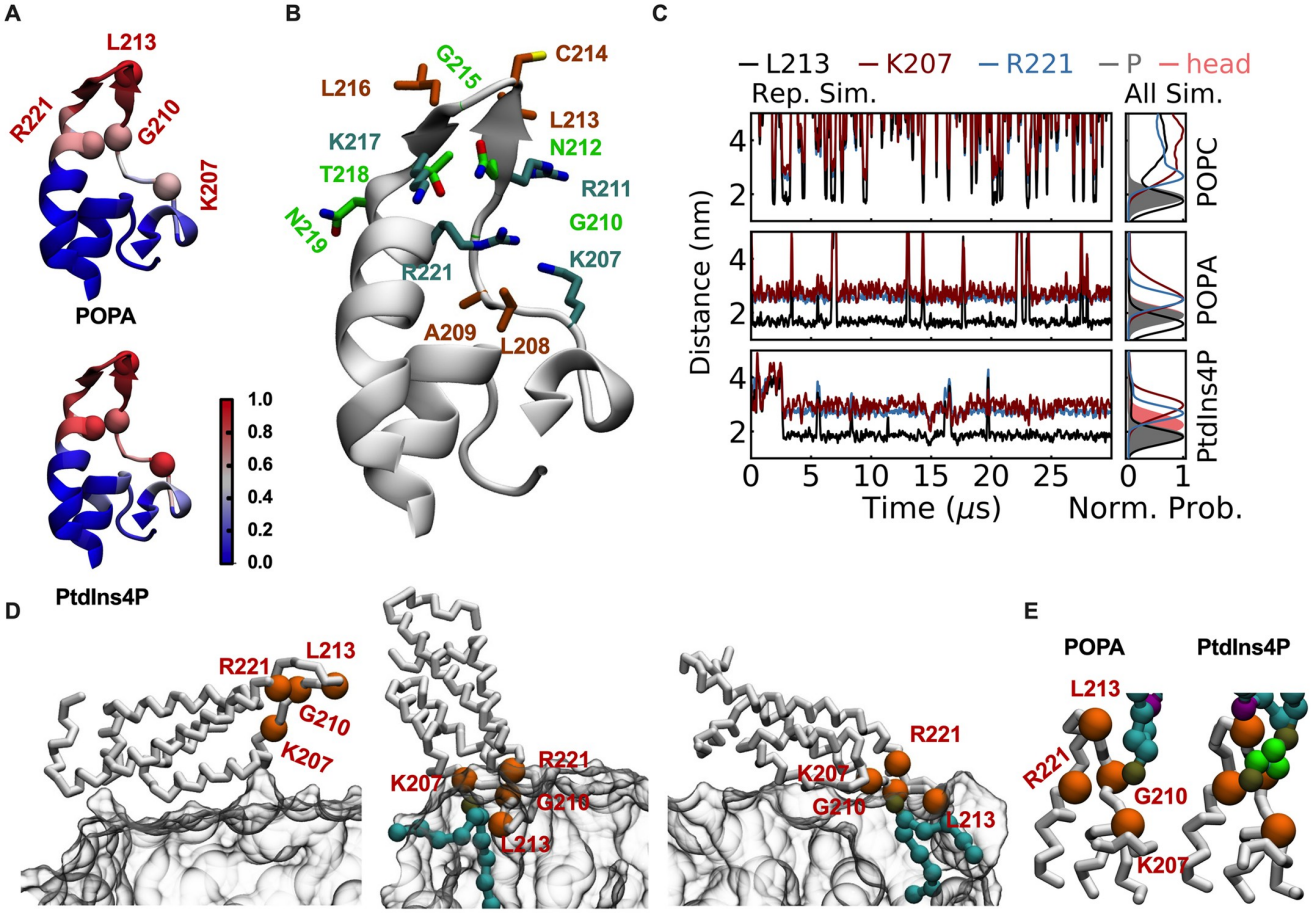
Interactions between BepE-BID1 and phospholipids revealed by coarse-grained molecular dynamics simulation. (A) Ratio of contacts between each protein residue and the lipids in the bilayer. (B) Residues of the HOOK subdomain. Positive residues are shown in cyan, hydrophobic residues in orange, while the other residues in green. (C) Distance between the geometric centers of L213, K207 and R221 and the geometric center of the bilayer. Left panel: distances as a function of simulation time in representative simulations; Right panel: distributions of the distances in all parallel simulations. The corresponding distributions of the phosphate group of POPC and the headgroups of the anionic phospholipids (phosphate group in the case of POPA and inositol-4-phosphate in the case of PtdIns4P) are also shown. Note that these two distributions overlap in the case of POPA. (D) Representative conformations for BID approaching membrane surface (the left panel) and binding to the bilayers (the middle and right panels). The POPA bound to BID is also shown. (E) The most abundant conformations for POPC and PtdIns4P bound to the HOOK subdomain. The coarse-grained beads representing the phosphate group are shown in brown, those representing the inositol of PtdIns4P in green and the beads representing the lipids tails are in cyan. See S4 Fig for other possible binding conformations.

We next employed coarse-grained molecular dynamics simulations to elucidate the binding mode between phospholipids and Beps. We simulated both pure POPC bilayers and mixed bilayers composed of 70% POPC and 30% POPA or PtdIns4P to explore their interactions with *Bhe* BepE-BID1. Notably, stable binding of BID domain to the mixed bilayers was observed, contrasting with the lack of binding to pure POPC bilayer. The ratio of contacts between each protein residue and the membranes suggested that BID bound to bilayers by the HOOK subdomain (see the S1 Movie and Fig 3A). The HOOK subdomain is composed of a short 3_10_ helix (η1) and a β hairpin [18], and forms a groove decorated with positively charged residues (theoretical isoelectric point value of 10.07). The significance of this positively charged groove is emphasized by the observation that BID domains from other Beps consistently exhibit a similar positively charged groove within their HOOK subdomains. This consistency suggests that the positively charged groove is a conserved feature among Bep BID domains, potentially serving as a crucial interaction interface for these proteins (S3 Fig). As shown in Fig 3B, the apical region of the HOOK subdomain features a unique arrangement of hydrophobic residues (L213 and L216) flanked by positively charged residues (K207, R211, K217 and R221). When BID bound to bilayers, the hydrophobic residues were inserted into the bilayer to interact with the fatty acyl chains of phospholipids while the positively charged residues located at the membrane surface interacted with the lipid headgroups (Fig 3C). In our simulations, BID approached the bilayers by its α-helices nonspecifically, and once it attached to the bilayer surface, the HOOK subdomain was attracted by the charged lipids and inserted into the bilayer quickly (Fig 3D). Membrane bound BID domain rotated and vibrated on the bilayers, with a tilting angle ranging between 40° and 80° (Figs 3D and S4). For each mixed bilayer, we clustered the conformations of the BID domain bound lipids to investigate their interaction mode in simulations. POPA and PtdIns4P showed consistent binding site and interaction mode. The binding site involved positively charged residues (K207, R211, K217 and R221) and uncharged G210. The negatively charged headgroups were located close to G210, R221 or K207 in the most abundant clusters (Figs 3E and S4).

In line with experimental observations, multiple parallel simulations also showed less affinity of BID to PtdIns4P than to POPA, as indicated by the additional local maximum at ~15° of the BID tilting angle distributions (S4 Fig, corresponding to a BID approaching the membrane surface shown in Fig 3D). Note that the hydrophobic residues of the HOOK subdomain inserted more deeply into the bilayers in POPA simulations than in PtdIns4P. The negatively charged phosphate group from PA, owing to its small size, is mostly buried within the hydrophobic interior of lipid bilayer. Therefore, deeper insertion into the bilayer is a probable prerequisite to stabilize PA-protein binding. We also hypothesize that the “less insertion” of hydrophobic residues may contribute to the weakened affinity of BID domain to PtdIns4P containing bilayers, although systematic estimation of its effect on the free energy of protein binding is out of the scope of this work.

**Fig 4:**
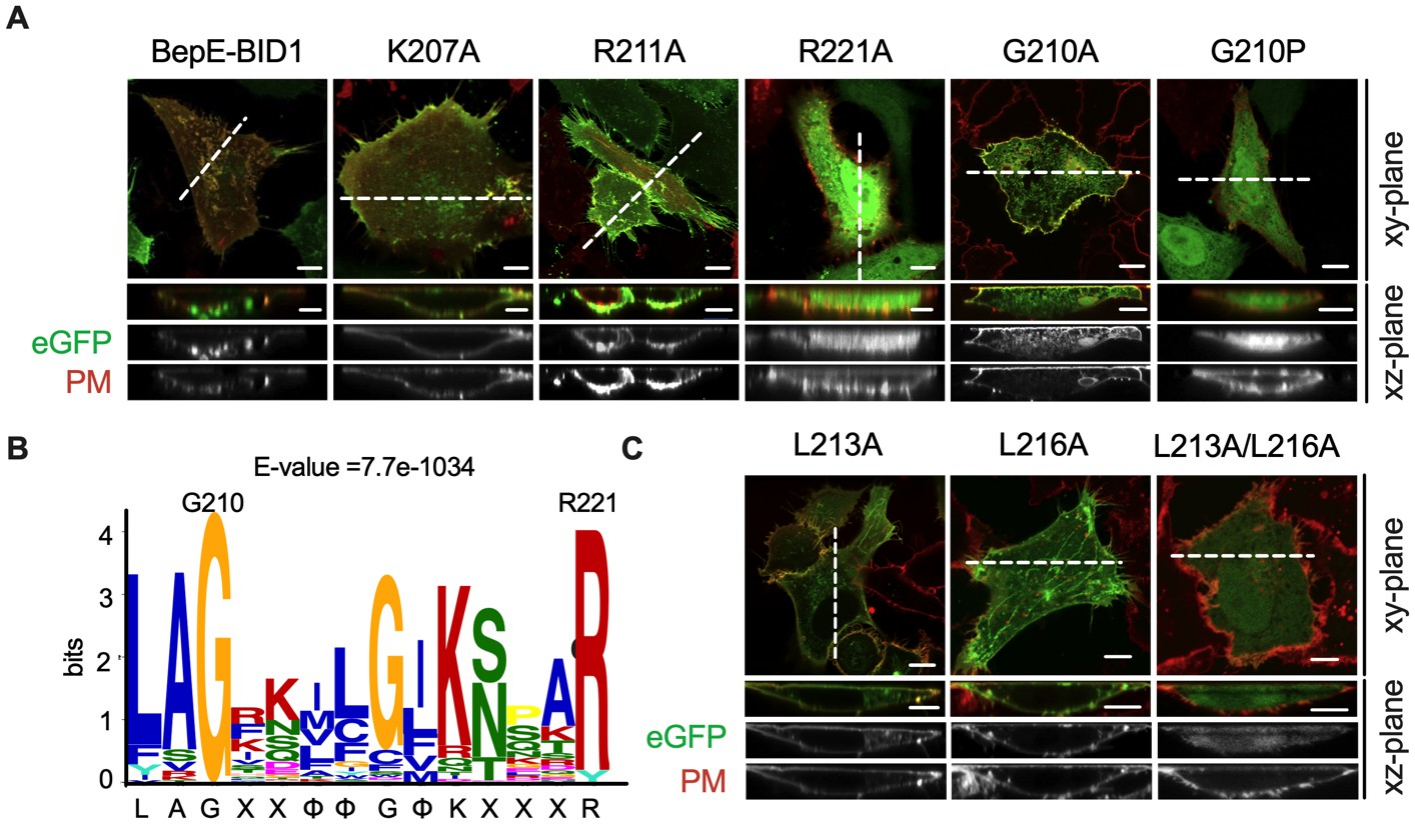
Critical residues determine the anchorage of BepE-BID at the plasma membrane. (A) HeLa cells were transfected with plasmids encoding BepE-BID1-wild type, BepE-BID1-K207A, BepE-BID1-R211A, BepE-BID1-R221A and BepE-BID1-G210P fused with eGFP tag. The plasma membrane is labeled in red (DiD). (B) Signature sequence of the PABM summarized from a sequence alignment of 176 BID domains. The MEME server [57] was used for conserved motif analysis of *Bartonella* effector proteins. The multi-sequence alignments of PABM from different *Bartonella* species are presented in the S1 Text. (C) Mutants of BepE-BID1-L213A, L216A and double site mutation were expressed into HeLa cells with eGFP tag. The plasma membrane is labeled in red (DiD). Representative images acquired in xy-plane and xz-plane. Scale bars = 10 μm.

Consequently, interacted residues K207, R211, G210 and R221 were individually mutated to alanine, and the mutants were transfected into HeLa cells. The R221A mutation resulted in cytosolic localization of BepE-BID1, G210A mutation partially disturbed the plasma membrane anchorage, whereas the other mutations had minimal effect on plasma membrane localization of BepE-BID1 (Fig 4A). Sequence alignment identified a conserved binding motif within the BID domain with a signature sequence of LAGxxΦΦGΦKxxxR (x represents any amino acid and Φ represents any hydrophobic amino acid) (Fig 4B). R221 was well conserved in all tested Beps, only some Bep6-BIDs from *B*. *rochelima* had a tyrosine residue instead of this arginine. Notably, we also observed that the position of G210 in the HOOK subdomain is invariable across all BID domains. Given that glycine cannot form a hydrogen bond with a phospholipid headgroup due to its uncharged nature, we assumed that the conserved glycine played a role in maintaining the conformation of the HOOK subdomain. Since the G210A (small side chain) mutation had a partial effect on subcellular localization of BID domain (Fig 4A), mutation of glycine to proline (contains a larger side chain) was constructed. The results showed that the G210P variant completely delocalized the BID domain from the plasma membrane to the cytosol (Fig 4A). By using homology modeling, we predict that the G210P substitution caused a structural alteration in the HOOK subdomain, specifically resulting in the steric hindrance of the positively charged groove, thus impeding the binding with phospholipids (S5 Fig). In order to confirm the effect of membrane insertion on BID localization, mutation of L213 and L216 to alanine (weaker hydrophobicity) was introduced. Results confirmed that L213A mutation caused partial disassociation of BID from plasma membrane to the cytosol, while minimal effect of L216A on the plasma membrane anchorage was observed. Additionally, the combined L213A/L216A substitutions induced a complete disassociation of BID from plasma membrane (Fig 4C).

### BID domain localization at the plasma membrane in a PA-dependent manner

**Fig 5:**
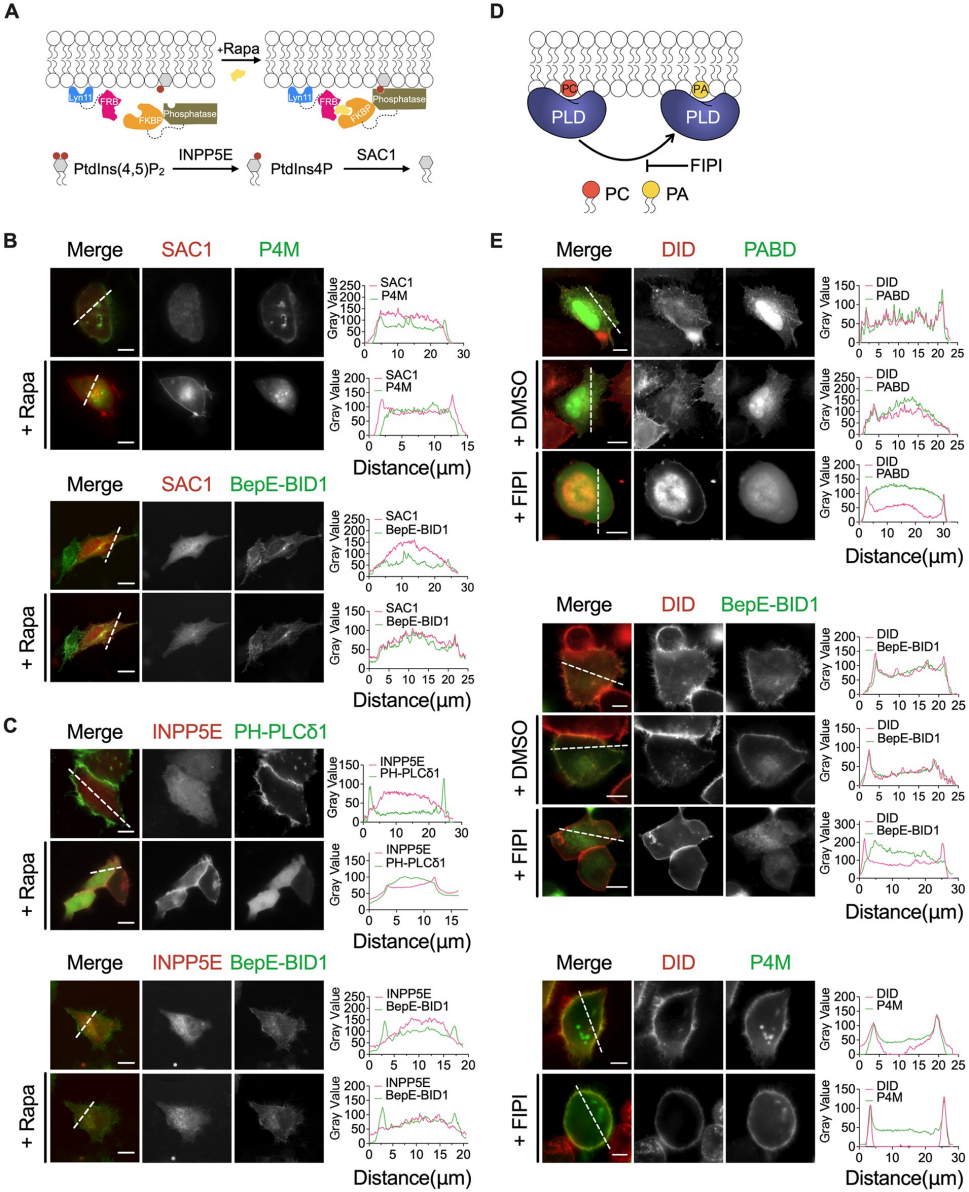
PA guides the plasma membrane association of BepE-BID1. (A) Schematic diagram showing the recruitment of the phosphatase to the plasma membrane using the FKBP/FRB rapamycin-inducible dimerization system (FKBP, 12-kDa FK506 binding protein; FRB, FKBP-rapamycin binding domain). (B) Representative images of shift in localization of BepE-BID1 and PtdIns4P probe P4M in HeLa cells upon plasma membrane recruitment of SAC1 induced by 1 μM rapamycin. (C) Representative images of shift in localization of BepE-BID1 and PtdIns(4,5)P_2_ probe PH-PLCδ1 in HeLa cells upon plasma membrane recruitment of INPP5E induced by 1 μM rapamycin. Fluorescence intensity was plotted along the lines. Scale bars, 10 μm. (D) FIPI inhibits the generation of PA by inhibiting the activity of phospholipase D (PLD). (E) Representative images of BepE-BID1, PABD and P4M in HeLa cells by stimulation with the PLD inhibitor FIPI for 2 hours. Fluorescence intensity was plotted along the lines. Scale bars = 10 μm.

To pinpoint the BID binding phospholipid that directs its plasma membrane localization, we co-transfected BepE-BID1 or corresponding phospholipid biosensors with Lyn11-FRB (rapamycin-binding domain of mTOR), alongside mRFP-FKBP (FK506 binding protein 12)-INPP5E (dephosphorylates PtdIns(4,5)P_2_ to PtdIns4P) or SAC1 (dephosphorylates PtdIns4P to PtdIns), which can be recruited to the plasma membrane in response to rapamycin [19,20] (Fig 5A). Recruitment of the phosphatase to the plasma membrane led to the dissociation of the PtdIns(4,5)P_2_ biosensor PH-PLCδ1 and the PtdIns4P biosensor P4M. However, BepE-BID1 localization remained unaffected upon depletion of these two phosphoinositides (Fig 5B and 5C), indicating that PtdIns4P and PtdIns(4,5)P_2_ were dispensable for BID domain’s plasma membrane localization. Subsequently, we introduced FIPI (5-Fluoro-2-indolyl des-chlorohalopemide), a PLD1 and PLD2 inhibitor blocking PA synthesis from plasma membrane-anchored PC [21] (Fig 5D), into HeLa cells expressing BepE-BID1. As anticipated, depletion of PA resulted in complete dissociation of BID domain from the plasma membrane to the cytosol, as evidenced by observations with the PA biosensor Spo20P-51-91 (Fig 5E). Notably, depletion of PA by FIPI had no effect on the plasma membrane localization of the PtdIns4P biosensor P4M (Fig 5E). These findings collectively support the conclusion that the localization of Beps at the plasma membrane is directed by the presence of PA.

### The plasma membrane anchorage of BID domain determines stress fiber formation induced by BepC

**Fig 6:**
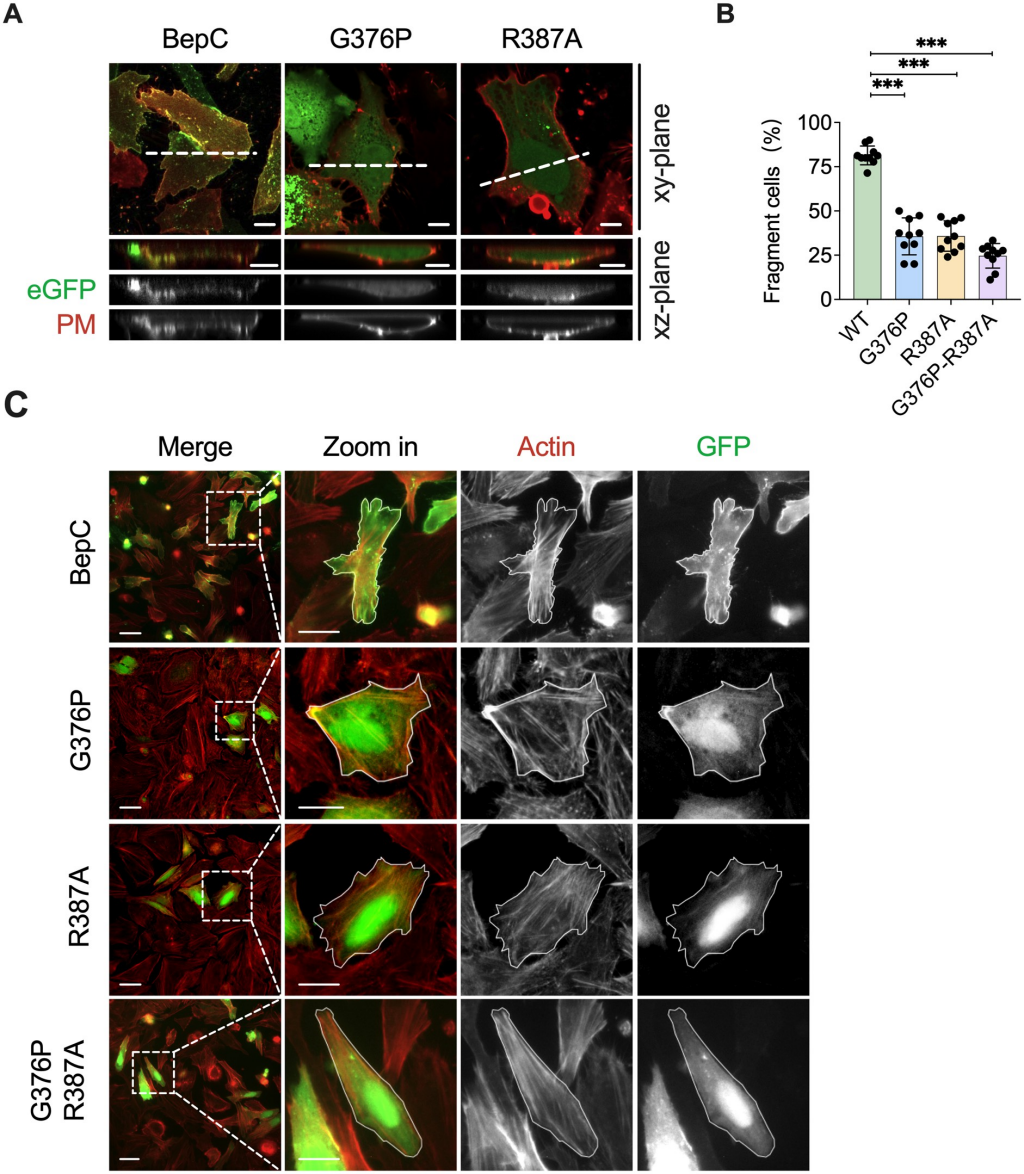
Anchorage of BID domain at plasma membrane determines stress fiber formation and cell fragmentation induced by BepC. (A) HeLa cells were transfected with BepC-wild type, BepC-G376P, or BepC-R387A fused with eGFP-tag. The plasma membrane is represented in red (DiD). Representative images acquired in xy-plane and xz-plane. (B) Cell fragmentation was quantified by manually analyzing 10 randomly selected visual fields. One-way ANOVA with multiple comparisons test was used. “***” p < 0.001. All experiments were performed more than three times independently, and representative data are shown. Values shown are means ± SD. (C) HeLa cells were transfected with plasmids encoding BepC-wild type, BepC-G376P, BepC-R387A or BepC-G376P-R387A fused with eGFP-tag. 24 hours after transfection, cells were fixed and stained with rhodamine phalloidin, followed by fluorescence microscopy analysis. Scale bars = 10 μm.

Previous investigations have established that the FIC domain of BepC binds to microtubule-localized GEF-H1 and tethers it to the plasma membrane *via* the BID domain. This interaction leads to the formation of a large amount of stress fibers, which are required for invasome structure formation through the GEF-H1/RhoA/Rock pathway [12,13]. In our study, we constructed BepC variants with individual and combined mutations G376P and R387A (corresponding to sites G210 and R221 of BepE-BID1 in the full-length protein of BepC). HeLa cells transfected with the corresponding mutant constructs were observed using confocal microscopy. Instead of localizing to the plasma membrane, these mutant variants were only found in the cytosol (Fig 6A). Furthermore, BepC mutants exhibited a significant reduction in stress fiber formation and cell fragmentation (Fig 6B and 6C). These findings underscore a robust correlation between the plasma membrane localization of Beps and their physiological roles in host cells.

### Functional significance of the cytosolic localization of BepD

**Fig 7:**
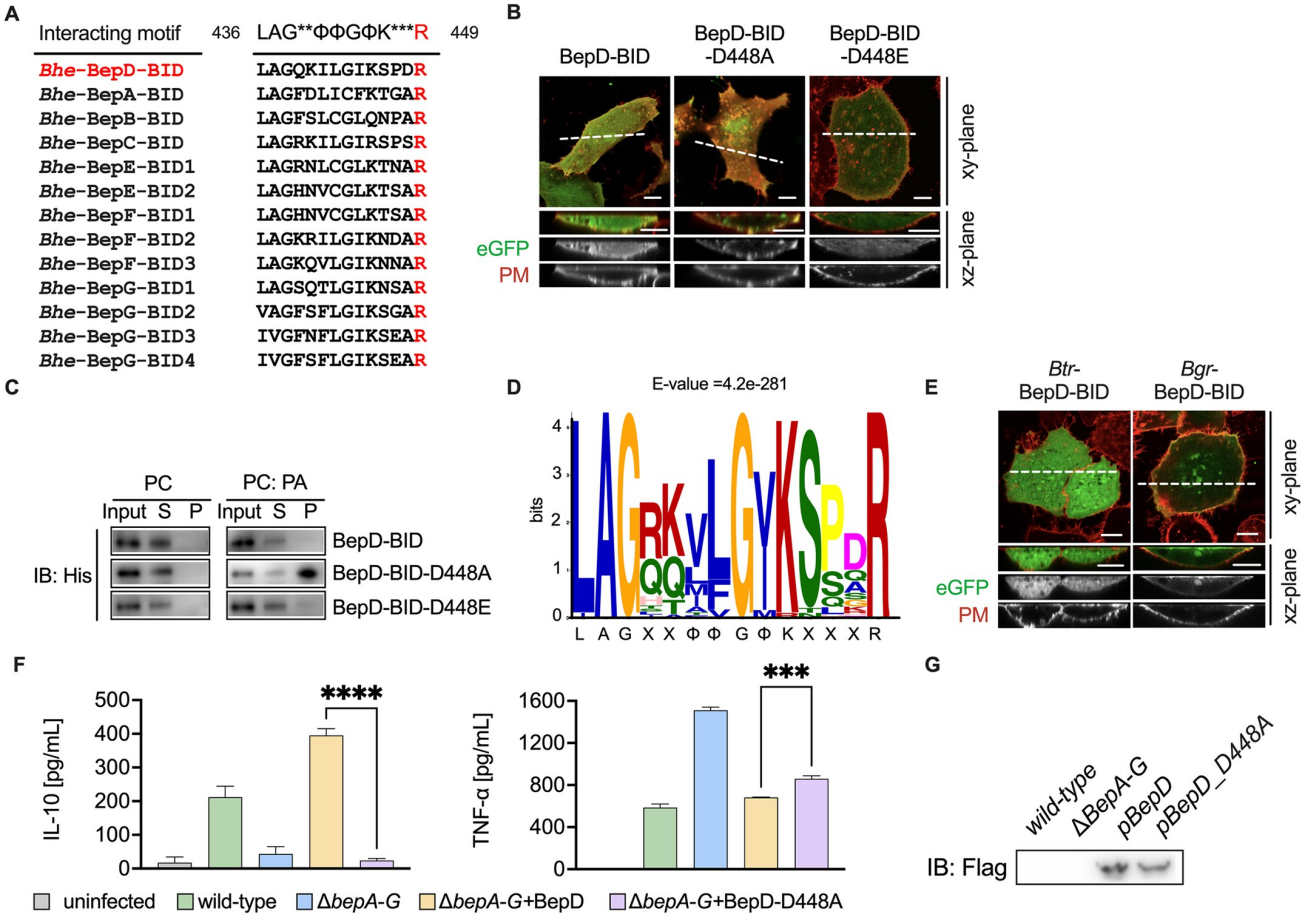
Cytosolic localization is required for BepD to modulate host innate immunity. (A) Sequence alignment of the phosphatidic acid binding motif of BID from *Bhe* effectors. Conserved arginine is highlighted in red. (B) HeLa cells were transfected with plasmids encoding BepD-BID, BepD-BID-D448A and BepD-BID-D448E fused with eGFP tag. (C) His-tagged BepD-BID, BepD-BID-D448A, and BepD-BID-D448E were incubated with liposomes either containing 100% PC or 90% PC plus 10% PA. Following ultracentrifugation of the liposomes, anti-His antibodies were used to detect BIDs in the pellet (P; bound to liposomes) or the supernatant (S; unbound to liposomes) fraction of the samples. (D) Signature sequence of the PABM summarized from a sequence alignment of 37 BepD-BID domains. The multi-sequence alignments of PABM from different BepD are presented in the S1 Text. (E) BepD-BID from *Btr* and *Bgr* fused with eGFP tag were expressed into HeLa cells. (F) RAW 264.7 macrophages were infected at MOI = 100 with *Bhe* wild type, the Δ*bepA-G* mutant, Δ*bepA-G* strains producing wild-type flag-tagged BepD or BepD-D448A mutant variant. At 24 hours post-infection, secreted IL-10 and TNF-α was quantified by ELISA. Data were analyzed by t test. “****” p < 0.0001. All experiments were performed more than three times independently, and representative data are shown. Values shown are means ± SD. (G) Δ*bepA-G* strains expressing wild-type flag-tagged BepD and mutant BepD-D448A were harvested from TSA plate supplemented with 10% defibrinated sheep blood. Protein production was assessed using western blotting after incubation in M199s medium. Representative images acquired in xy-plane and xz-plane. Scale bars = 10 μm.

Despite exhibiting structural similarities with BID domains from other effectors (S3 Fig), BepD-BID predominantly localizes to the cytosol, with a minor fraction associated with the plasma membrane (Fig 7B). Sequence alignment analysis revealed the presence of a negatively charged aspartic acid (D448) preceding the conserved arginine R449 (corresponding to R221 of BepE-BID1), where an uncharged amino acid typically exists in other BID domains (Fig 7A). The presence of the negative charge likely disrupts the electrostatic binding of R449 with PA, leading to the cytosolic localization of BepD-BID. To test this hypothesis, we generated D448A or D448E variants of BepD-BID and transfected them into HeLa cells. As expected, D448A exhibited a complete localization to the plasma membrane, while D448E remained in the cytosol (Fig 7B). Although protein-lipid overlay assay confirmed that BepD can bind to PA, BepD-BID and BepD-BID-D448E mutant exhibit a weak binding to PA liposomes, whereas BepD-BID-D448A mutation had an enhanced affinity to PA liposomes (Fig 7C). Sequence alignment of BepD from various *Bartonella* species also identified frequent mutations to aspartic acid or glutamic acid in BepD homologs (Fig 7D). Immunostaining confirmed that mutation to aspartic acid resulted in cytosolic localization of *Btr*-BepD-BID, while the native BepD-BID from *B*. *grahamii* (*Bgr*) remained localized at the plasma membrane (Fig 7E).

BepD has been reported to inhibit TNF-α secretion but to increase IL-10 secretion from infected macrophages, establishing an immune-silent environment [22]. To investigate the importance of cytosolic localization in the immunomodulatory effects of BepD, RAW264.7 cells were infected with the *Bhe* wild type or isogenic mutants for 24 hours at a multiplicity of infection (MOI) of 100. Compared to the low level of IL-10 secretion observed with the Bep-deficient strain Δ*bepA-G* (<50 pg/mL), infection with the Δ*bepA-G* strain complemented with wild-type BepD resulted in a significant increase in IL-10 secretion (Fig 7F and 7G). Meanwhile, infection with the Δ*bepA-G* strain induced significant TNF-α secretion in RAW264.7 cells, while translocation of wild-type BepD impaired TNF-α secretion (Fig 7F and 7G). However, disruption of the shift in localization from the plasma membrane to the cytosol of BepD by the D448A mutation failed to promote IL-10 secretion and inhibit TNF-α secretion (Fig 7F and 7G).

## Discussion

Eukaryotic cells are equipped with a dynamic membrane system comprising the plasma membrane and the membranes of intracellular organelles [23]. In the event of bacterial infection of the host cells, effector proteins can bind to diverse lipid molecules, thereby localizing to different organelle membranes. Subsequently, these bacterial effectors disrupt various cellular physiological activities upon their membrane localization.

To date, the subcellular localization of bacterial effectors at the inner leaflet of the plasma membrane has traditionally been associated with their interaction with phospholipids such as PtdIns4P or PtdIns(4,5)P_2_ [24–26]. While previous studies have shown that some bacterial effectors can directly bind to PA, there has been limited evidence supporting the dependence of bacterial effector localization on PA binding [3,27–30]. Our study adds to this understanding by demonstrating that interaction with PA is also effective in directing the localization of bacterial effectors to the inner leaflet of the plasma membrane. PA plays a crucial role in cellular function due to its involvement in glycerophospholipid synthesis and its numerous signaling functions within the cell, including cell proliferation, vesicular trafficking, signal transduction, and cytoskeleton rearrangement [31]. Structurally, the PA molecule consists of a glycerol backbone, two hydrophobic fatty acid side chains, and an anionic phosphomonoester headgroup, which is known to bind to proteins through interactions with lysine or arginine residues [32]. PA is found in various cellular membranes, including mitochondria, the ER, and other organelles [31]. The electrostatic/hydrogen bond switch mechanism illustrates how the formation of hydrogen bonds with positively charged residues can lead to the deprotonation of the phosphate group of PA, increasing its negative charge from -1 to -2 and favoring the stabilization of protein-PA interactions [31]. Additionally, increasing the pH or bilayer concentration of phosphatidylethanolamine (PE), which can form hydrogen bonds with PA due to its primary amine, results in PA carrying two negative charges [27]. The higher ratio of PE to PC in the plasma membrane compared to the ER and other organelles facilitates the anchoring of PA-binding proteins at the plasma membrane [33,34]. Furthermore, PA preferentially accumulates at sites of negative membrane curvature due to its small headgroup moiety, enabling PA-binding proteins to localize at specific regions within the plasma membrane where they execute their specialized roles [31,35]. Almost half of *Bartonella* effectors involve in the formation of “Invasome” to invade endothelial cells [14,36,37]. Negative membrane curvature on either side of positive membrane curvature caused by *Bartonella* aggregates is beneficial for local positioning and concentration of Beps, so that BepC can induce stress fiber formation *in situ* to facilitate engulfment of *Bartonella* aggregates, together with BepF and BepG. These specific characteristics of PA explain why *Bartonella* effectors localize at the plasma membrane rather than to other organelles through binding with PA, and why they preferentially select PA over PtdIns(4,5)P_2_ for anchoring at the plasma membrane.

Local positioning of effectors through binding to PA is beneficial for *Bartonella* to modulate the dynamics of the host cell cytoskeleton. Notably, BepD stands out as the sole effector reported to regulate host immune reactions. BepD is phosphorylated by Src-family kinases in host cells and then plays role as a signaling hub for STAT3 activation through recruitment of c-Abl, leading to the upregulation of IL-10 and the inhibition of TNF-α secretion [23]. To ensure sufficient contact with cytosolic, unphosphorylated STAT3, BepD undergoes a critical transition from its membrane-bound state to the cytosol. This transformation is orchestrated by a specific substitution at the -1 position preceding the conserved arginine residue, where alanine is replaced with aspartic acid. Whether mutations of critical amino acids that determine membrane anchorage also exist in other Beps and their biological relevance should be investigated in further studies.

Many mammalian proteins have been reported to recognize PA, including protein kinases [38], phosphatases [39], cAMP-specific phosphodiesterase [40], and transcription factors [41]. However, there is no clearly defined globular domain to which phosphatidic acid-binding activity can be ascribed in contrast to other known lipid-binding domains, such as Pleckstrin Homology (PH), Phox (phagocyteoxidase) Homology (PX), Fab 1-YOTB-Vac 1-EEA1 (FYVE) [32]. The regions implicated in PA-binding tend to comprise short stretches enriched with polybasic amino acids [35]. In this study, we have discovered a conserved PA binding motif in *Bartonella* Beps characterized as LAGxxΦΦGΦKxxxR, which is similar to other bacterial phospholipid binding domains by the presence of polybasic residues and at least one hydrophobic residue [42]. PA-binding region in BID domain consists of a short 3_10_ helix (η1) and a β hairpin to form a "Hook”-like structure. Hydrophobic amino acid at the head region of HOOK subdomain inserts into lipid bilayer so that positively charged residues can interact with negatively charged phosphate. Electrostatic-, hydrogen bond-, and hydrophobic interactions acting in concert will likely be the key features of PA-binding domain to allow for a highly specific localization.

PA-binding domain derived from the yeast sporulation protein Spo20p is the most widely used probe for studying PA dynamics. Interestingly, different PA-binding domains are often found in different subcellular compartments. For example, the Spo20p-PABD generally accumulates in the nucleus and at the plasma membrane in mammalian cells [43], whereas the yeast protein Opi1p (Opi1p–PABD) shuttles between the ER and the nucleus [44]. Some evidence suggests that these different PA-binding domains may sense distinct pools of PA within specific bilayer environments [32], or these probes might have other phospholipid targets on the membrane besides PA. Therefore, the specific PA-binding characteristic and plasma membrane localization of the BID domain enable it to serve as a potent biosensor for studying the dynamics of plasma membrane resident-PA in mammalian cells.

## Materials and methods

### Bacterial strains, cell lines and growth conditions

The bacterial strains used in this study are listed in S3 Table. *Escherichia coli* strains were cultured in lysogeny broth (LB) or on solid agar plates (LA), supplemented with appropriate concentrations of antibiotics at 37°C. *Bartonella henselae* strains were inoculated on Tryptic soy agar plates (TSA, BD Biosciences) with 10% defibrinated sheep blood at 35°C and 5% CO_2_, then subcultured for three days on fresh TSA plates. HeLa cells (human cervix carcinoma cells, ATCC: CCL-2), HEK293T (ATCC: CRL-1573) and RAW 264.7 (murine macrophage cell line, ATCC TIB-71) cells were cultured in Dulbecco’s modified Eagle’s medium (DMEM, Gibco) supplemented with 10% fetal bovine serum (Meiluncell), penicillin (100 units/mL), and streptomycin (100 μg/mL) at 37°C with 5% CO_2_. HUVECs (human umbilical vein endothelial cells) were cultured in endothelial cell medium (OriCell) at 37°C with 5% CO_2_.

### Immunofluorescence

HeLa cells were transfected with plasmid DNA using the Effectene transfection reagent (Qiagen) according to the manufacturer’s protocol. HeLa cells were transfected for 24 hours with 800 ng DNA in glass-bottom 35 mm confocal dishes. Where indicated, cells were washed with PBS 3 times, then fixed with 4% paraformaldehyde (PFA) for 20 minutes, after that plasma membrane were stained with 10 μM 1,1’-Dioctadecyl-3,3,3’,3’-Tetramethylindodicarbocyanine,4-Chlorobenzenesulfonate Salt (DiD’ solid, Beyotime), filamentous actin (F-actin) were stained with 0.1 μM Rhodamine phalloidin (ABclonal). Cells infected with *Bartonella* were fixed and permeabilized using saponin, which selectively permeabilizes mammalian cell membranes but not bacterial cell membranes, and then stained with Flag antibody (ACE Biotechnology) and Rhodamine phalloidin (ABclonal). Cells were imaged by an epi-fluorescence microscope (EVOS FL-2) or Nikon eclipse Ti2 AX confocal microscope (Nikon).

### Bacterial infection

The day before infection, 2.5 × 10^5^ cells, were seeded per well in 24-well plates. *Bhe* was harvested from TSA plates after three days of culture, mixed by vortex, and incubated in M199s overnight at 37°C with 5% CO_2_. The cells were infected with bacteria at an MOI of 300 (HUVECs) or 100 (RAW264.7) in M199s (HUVECs) or DMEM (RAW 264.7) supplemented with 100 μM isopropyl-β-D-thiogalactoside (IPTG). The plate was then centrifuged at 130 x g for 10 minutes and incubated at 37°C with 5% CO_2_.

### Recombinant protein production and purification

Expression of full-length *Bartonella* effector proteins was unsuccessful in laboratory strains of *E*. *coli*, likely due to the absence of specific chaperones that are required for their solubility and stability. Therefore, expression in *Bartonella* Δ*bepA-G* harboring *pANT4*-Beps or inducible *pBZ485-*BepC (constitutive expression of BepC causes toxicity to *Bartonella*) plasmids were used for recombinant protein production. *Bhe* was grown on TSA plates at 37°C and 5% CO_2_ for 3 days and then incubated in the M199s overnight. Bacteria were harvested by centrifugation at 4000 g for 3 minutes and washed twice with PBS at 4°C. The cell pellet was resuspended in Tris-buffered saline (TBS) containing lysozyme and protease inhibitor, incubated for 5 minutes. Then, bacteria were lysed by ultrasonic treatment and the lysates were centrifuged at 6000 g for 20 minutes. The clear supernatant was collected and mixed with Anti-DYKDDDDK G1 affinity resin (Genscript) overnight. Resin was then washed 3 times with TBS and bound protein was eluted with TBS containing 250 μg/mL 3x Flag peptide (Beyotime) for 2 hours. Purified proteins were verified by western blot and stored at -80°C.

BID1 of *Bhe*-BepE was obtained from *E*. *coli* expression. *E*. *coli* grown in LB broth with 50 μg/mL kanamycin at OD_600_ value of 0.6. Then, 100 μM IPTG was added, and further incubated for 12 hours at 16°C. *E*. *coli* was lysed and clear supernatant was collected. His-tagged BID1 were purified using Ni-NTA agarose resin (Yeasen). Purified proteins were verified by western blot and stored at -80°C.

### Protein-lipid overlay assay

The binding of Beps to lipids was tested in protein-lipid overlay assays using membrane lipid strips (Echelon Biosciences). Lipid strips were blocked for 1 hour with PBS/0.1% Tween-20 (PBST) containing 4% BSA (PBST-BSA) before 2 hours incubation with control 3x Flag tag (2 μg/mL) or recombinant Beps fused with Flag tag (2 μg/mL) in PBST-BSA. Membranes were then washed with PBST 3 times, probed for 2 hours with mouse anti-Flag antibody in PBST-BSA, followed by 3 washes with PBST. The membranes were further incubated with HRP-conjugated anti-mouse IgG secondary antibody (Beyotime) in PBST-BSA for 1 hour and then visualized by Tanon-5200 Chemiluminescent imaging system.

### Preparation of liposomes and pull-down assay

Liposomes were prepared using the thin film hydration method. PC alone or PC with the lipids of interest (Echelon Biosciences) were mixed in chloroform at a molar ratio of 9:1 for a total lipid amount of 8 μM, while appropriate amounts of DSPE-PEG2000 and cholesterol were added to stabilize the liposome structure. After evaporation of the lipid solution, the thin lipid film was vacuum dried at 37°C. The thin lipid film was then resuspended in PBS and vortexed at 37°C to form a milky and uniform suspension. The resuspended lipid solution was sonicated in flask for 2 minutes using an ultrasonic probe at a frequency of 24 kHz and power of 60W. Subsequently, the resulting vesicles were extruded 11–21 times serially through 400nm, 200nm and then 100nm polycarbonate porous membranes using an Avanti mini extruder (Avanti Polar Lipids) to obtain the target liposomes. Hydrodynamic diameter and dispersibility of liposomes were detected by a Malvern Zetasizer Nano ZS90 instrument (Malvern). The morphologies of the liposomes were characterized *via* Cryo-TEM utilizing a Talos F200C G2 field emission transmission electron microscope (FEI-Thermo Fisher Scientifc).

Binding of BepE-BID1 with His-tag to liposomes was determined by liposomes pull down assay. The liposomes were prepared at 0.5 mg/mL and incubated with purified protein at 4°C for 30 minutes. Liposome-protein mixture was then pelleted by centrifuging at 4°C, at 100 000 g for 30 minutes, suspended in 1x LDS loading buffer and subjected to SDS-PAGE.

### Molecular interaction

Lipid/BepE-BID1 interactions were detected using the metasurface plasmon resonance (MetaSPR) LifeDisc biosensors (Highly Sensitive) G10001 chip (XLement). According to the instructions, the BepE-BID1 protein was prepared into a solution of 25 μg/mL using CBS buffer. After adding 2 μL per well onto the chip surface, it was left to incubate and fix at 4°C for 8 hours. Next, blocking solution was added onto the chip surface and incubated at 37°C for 1 hour, followed by removal of the blocking solution and drying with nitrogen gas. The PC, PC: POPA or PC: PtdIns4P liposomes were diluted with HBS-ET buffer, then added into the sensor chip wells at a dose of 50 μL per well simultaneously using the 8-channel pipettor, and allowed to react for 4 minutes. After the reaction, the chip plate is tapped dry, and 150 μL per well of HBS-ET buffer was simultaneously added into the sensor chip wells using the 8-channel pipettor, and incubated for 8 minutes. Binding affinities (K_D_) were determined from concentration gradient experiments and by WeSPR 200 software (XLement).

### Immunoblot analysis

Samples were suspended in 1x LDS loading buffer (ACE Biotechnology) and boiled 10 minutes. After brief sonication, samples were loaded on 4–20% Bis-Tris gradient gels (ACE Biotechnology) and transferred onto PVDF membranes, which were blocked in 5% skim milk in TBS and probed with the indicated primary antibodies and HRP-labeled secondary antibodies at the concentrations indicated. The blots were detected using Tanon-5200 Chemiluminescent imaging system.

### Quantification of cytokines

Cell culture supernatants were analyzed by ELISA for IL-10 and TNF-α according to the manufacturer’s instructions (ABclonal). In brief, after washing the wells of 96-well plate, 100 μL sample was added and incubated for 2 hours at 37°C. The plate was then washed, and a biotin-conjugated antibody (1:100) was added to each well. The plate was incubated for 1 hour at 37°C, washed, and Streptavidin-HRP was added for 30 minutes at 37°C. The plate was then washed, and TMB substrate solution was added to each well for 15 minutes at 37°C. Finally, the reaction was stopped by adding the stop solution. The absorbance was measured at 450 nm and 570 nm. At least three independent experiments (n = 3) were performed with technical triplicates.

### Molecular dynamics simulations

Coarse-grained molecular dynamics simulations were performed to investigate the interactions between *Bhe*-BepE-BID1 (PDB ID: 4YK3) with pure POPC bilayers and mixed bilayers composed of POPC and negatively charged lipids (mole ratio of 7:3, 2 systems containing POPA or PtdIns4P, respectively). The bilayers were equilibrated for 0.1 μs before the BID protein was positioned randomly in the simulation box. 30 μs simulations were then performed, with 5 parallel simulations for each system. Each simulation system contained ~170 lipids, 1 protein, ~10000 coarse-grained water beads and necessary number of counterions to neutralize the simulation system. The accumulated simulations time is ~450 μs.

The Martini 3.0 force field [45] and the phosphatidylinositide lipid extension [46] were employed to describe the simulation system. The bilayers were constructed by the *insane*.*py* script [47]. The atomic structure of the protein was converted to coarse-grained model by the *Martinize* script, with the Gō-Martini model [48] applied to restrain its structure. The simulations were conducted by the GROMACS2021 [49] package with an integration timestep of 20 fs. The temperature was maintained at 310 K using the v-rescale thermostat [50] and a relaxation time of 1 ps. We applied semi-isotropic pressure coupling to maintain the pressure of the system at 1 bar, with the Berendsen barostat, [51] a relaxation time of 4.0 ps and a compressibility of 3×10^−4^. A cutoff value of 1.1 nm was used to turn off the van der Waals interactions. The electrostatic interactions were calculated using the same cutoff distance and a dielectric constant of 15, with the long range electrostatic interactions included by the reaction-field method [52].

We counted the ratio of contacts between each protein residue and the bilayer to investigate which portion of the protein interact with the membrane. Specifically, a residue was considered to have a contact with lipids in a frame if the distance between any of its coarse-grained bead and a lipid bead is smaller than a cutoff value of 0.7 nm. The ratio of contacts was defined as the number of contacts across the trajectory divided by the total number of frames. The ratio of contacts between each residue and the headgroups of the negatively charged lipids was also calculated to investigate the lipid binding site of the protein. We clustered the conformations of the protein bound lipids to study their interaction mode. First, we selected the negatively charged lipid whose headgroup (phosphate group in the case of PA and inositol-4-phosphate in the case of PtdIns4P) was closest to the geometric center of the “binding residues” (defined as K207, G210, R211, K217 and R221, i.e., the four positively charged residues and the glycine at the HOOK subdomain) in each frame. We then clustered the selected lipids based on the RMSD values of their headgroups which were calculated after superimposing the conformations based on the protein backbone beads. The clustering was performed using the gromos algorithm [53] with RMSD cutoff values of 0.3 nm (for POPA) or 0.6 nm (for PtdIns4P). We presented the three most abundant clusters, which accounts for ~30–65% of the total conformations depending on the specific case. In order to characterize the configurations of membrane bound BID, we calculated the tilting angle of BID in our simulations, which was defined as the angle between the axis of the protein and the normal direction of the bilayer. The axis of the protein was defined as the vector fitted based on the residues of the α-helices of BID using singular value decomposition method.

### Statistical analysis

GraphPad Prism 8.0 Software was used to perform all statistical analyses. Bar graph data are presented as mean ± SD. Statistical significance was determined by using one-way ANOVA with multiple comparisons test. p values less than 0.05 were considered to be statistically significant: * p < 0.05; ** p < 0.01; *** p < 0.001. The number of biological and technical replicates is stated in figure legends.

## Supporting information

S1 Fig Subclasses of BID from *Bartonella* effector Beps. Phylogenetic tree with the maximum likelihood method represents the multiple sequence alignment of the BID from different Beps. Ancestral tBIDx are colored in green, BIDx in blue and tBID(dark)/nt-BID1(light) in purple. This tree includes the species names and UniProt IDs.
(TIF)

S2 Fig Molecular dynamics simulation of interactions between BID domain and lipids. (A) Cryo-transmission electron microscope (Cryo-TEM) image of liposome (90% PC and 10% PA). Scale bar = 100 nm. (B) The hydrodynamic diameter distribution of liposomes dispersed in PBS was determined by dynamic light scattering (DLS).
(TIF)

S3 Fig Homologous modeling of the BID domain shows a conserved positively charged surface at the hook subdomain. The electrostatic potential of experimentally determined and modeled BID domain structures is depicted. Protein surfaces are colored according to electrostatic potentials, with red and blue indicating negative and positive potentials, respectively. The structures of BID domains were predicted with Alpha-Fold2.
(TIF)

S4 Fig Molecular dynamics simulation of interactions between BID domain and lipids. (A) Distance of the geometric center of L213 to the bilayer center and the tilting angle of BID relative to the normal of the bilayer plane. Left panel: the corresponding values as a function of simulation time in representative simulations; Right panel: the distributions of corresponding values in all parallel simulations. (B) The most abundant three clusters for the binding conformations of the negatively charged lipid headgroups to BID domain. The coarse-grained beads representing the phosphate group of negatively charged lipids are shown in brown. The beads representing the inositol of phosphatidylinositide lipids are shown in green.
(TIF)

S5 Fig Structural changes caused by mutation of glycine to proline in HOOK subdomain. (A) Overlay of *Bhe*-BepE-BID1 (green, PDB: 4YK3) and *Bhe*-BepE-BID1-G210P (pink). The predicted structures of *Bhe*-BepE-BID1-G210P were generated using Alpha-Fold2, and the structural alignment was performed using PyMol. (B) The electrostatic potential of *Bhe*-BepE-BID1 and *Bhe*-BepE-BID1-G210P. The protein surfaces are color-coded according to electrostatic potentials, with red representing negative potentials and blue representing positive potentials.
(TIF)

S1 Text The multi-sequence alignments. (DOCX)

S1 Movie Ratio of contacts between each protein residue and the bilayer. (MP4)

S1 Table Oligonucleotides used in this study. (DOCX)

S2 Table List and construction of all plasmids used in this study. (DOCX)

S3 Table Bacterial strains used in this study. (DOCX)

S4 Table The raw data file contains primary experimental data and statistical analysis for Figs 2B, 2D, 5B–5C, 5E, 6B, 7F and S2B. (XLSX)
