# Supplementary figures and images for: Anchorage of bacterial effector at plasma membrane via selective phosphatidic acid binding to modulate host cell signaling
Source: PLoS Pathog. 2024 Nov 12;20(11):e1012694. doi: 10.1371/journal.ppat.1012694 (PMC11556746; doi:10.1371/journal.ppat.1012694)

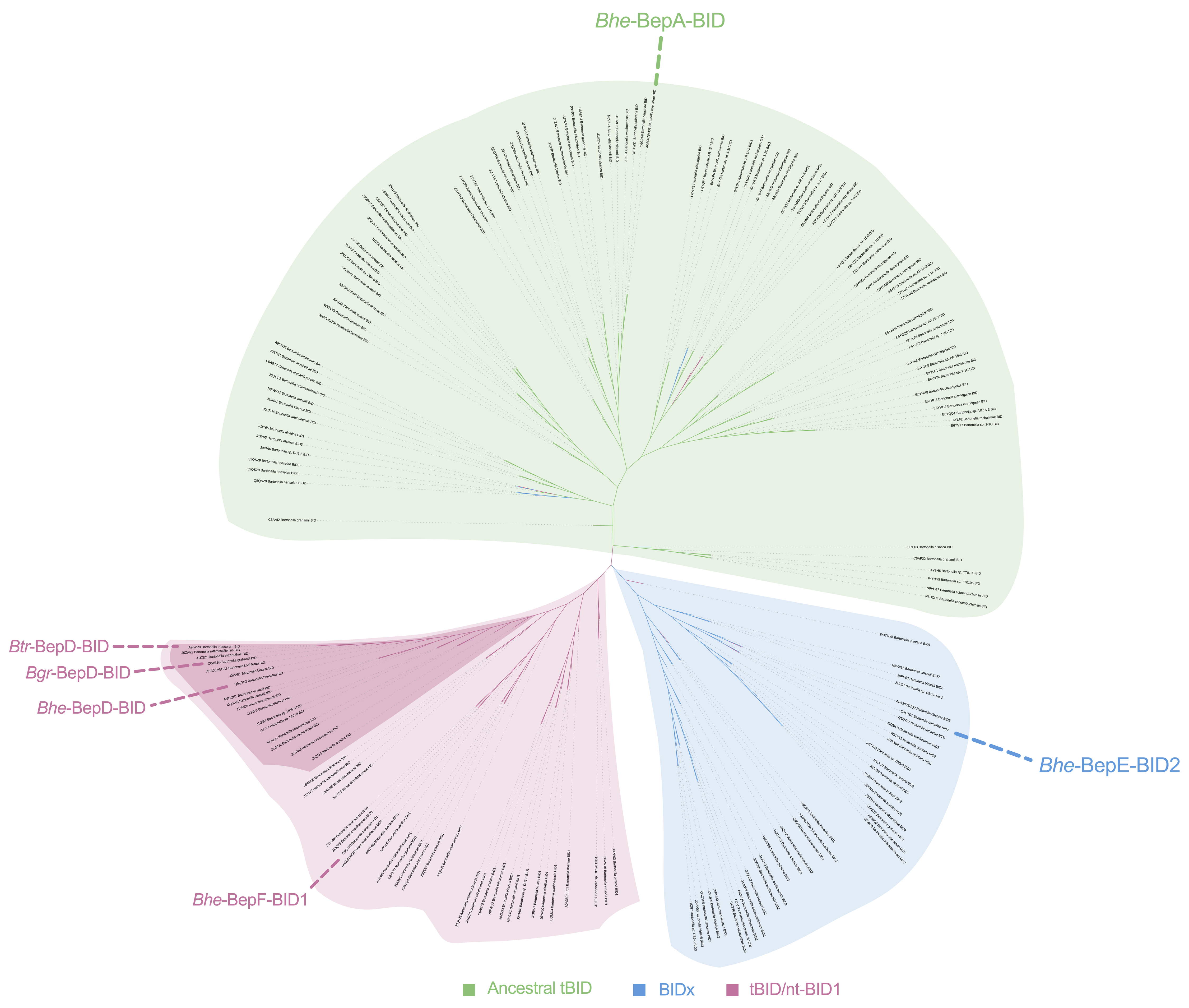

Supplement: S1 Fig — Phylogenetic tree with the maximum likelihood method represents the multiple sequence alignment of the BID from different Beps. Ancestral tBIDx are colored in green, BIDx in blue and tBID(dark)/nt-BID1(light) in purple. This tree includes the species names and UniProt IDs. (TIF) [file ppat.1012694.s001.tif]

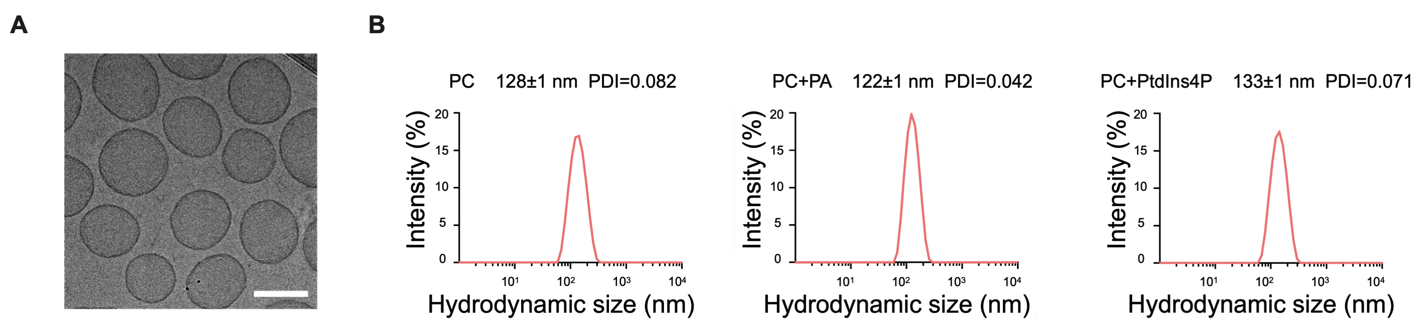

Supplement: S2 Fig — (A) Cryo-transmission electron microscope (Cryo-TEM) image of liposome (90% PC and 10% PA). Scale bar = 100 nm. (B) The hydrodynamic diameter distribution of liposomes dispersed in PBS was determined by dynamic light scattering (DLS). (TIF) [file ppat.1012694.s002.tif]

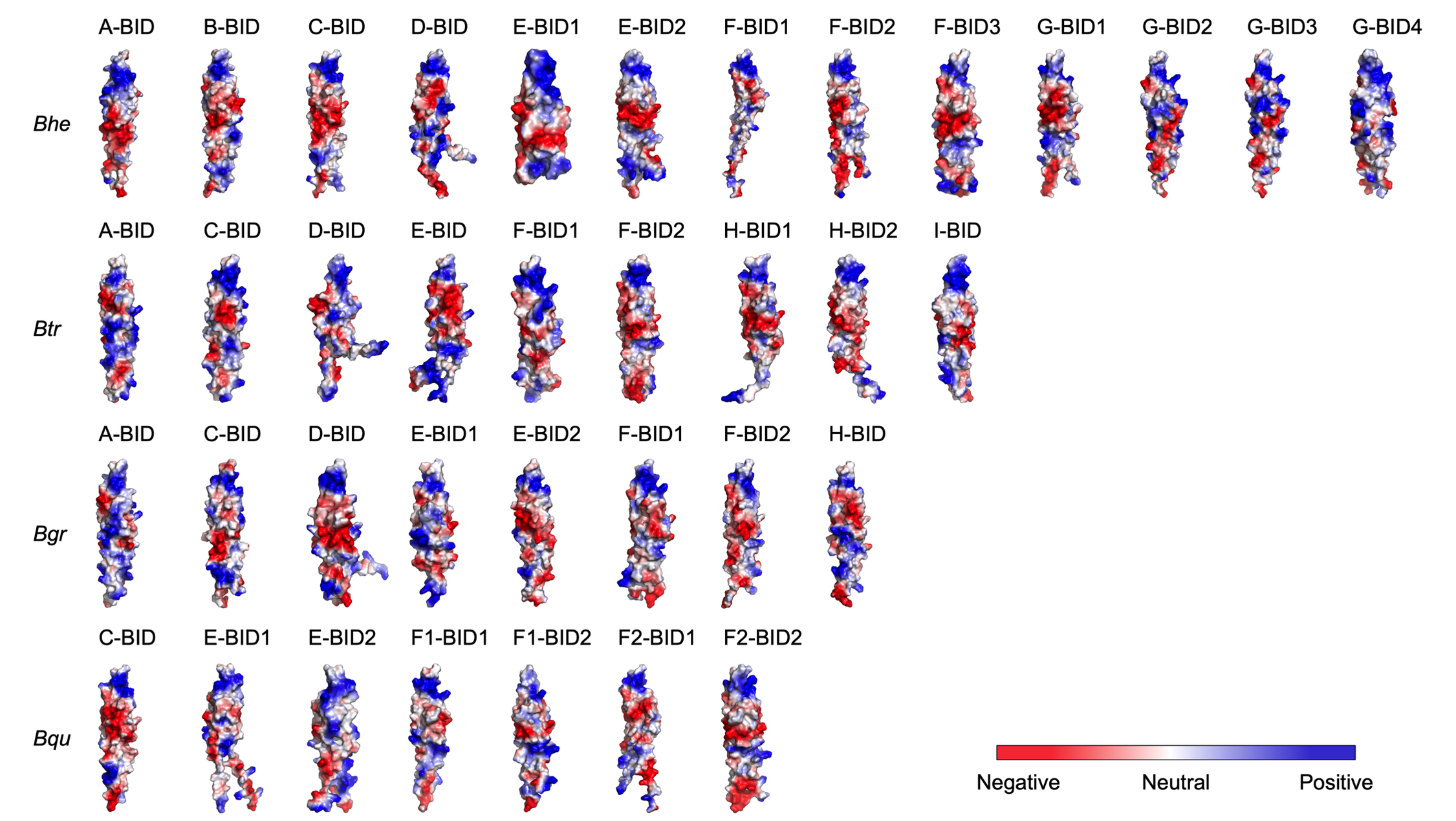

Supplement: S3 Fig — The electrostatic potential of experimentally determined and modeled BID domain structures is depicted. Protein surfaces are colored according to electrostatic potentials, with red and blue indicating negative and positive potentials, respectively. The structures of BID domains were predicted with Alpha-Fold2. (TIF) [file ppat.1012694.s003.tif]

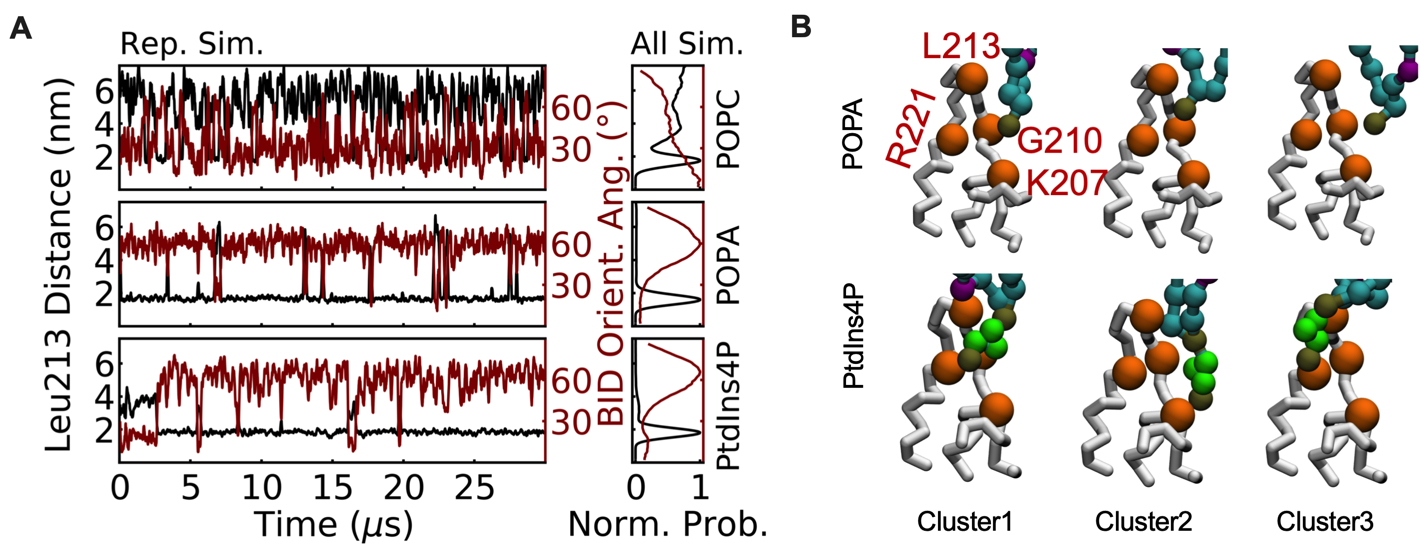

Supplement: S4 Fig — (A) Distance of the geometric center of L213 to the bilayer center and the tilting angle of BID relative to the normal of the bilayer plane. Left panel: the corresponding values as a function of simulation time in representative simulations; Right panel: the distributions of corresponding values in all parallel simulations. (B) The most abundant three clusters for the binding conformations of the negatively charged lipid headgroups to BID domain. The coarse-grained beads representing the phosphate group of negatively charged lipids are shown in brown. The beads representing the inositol of phosphatidylinositide lipids are shown in green. (TIF) [file ppat.1012694.s004.tif]

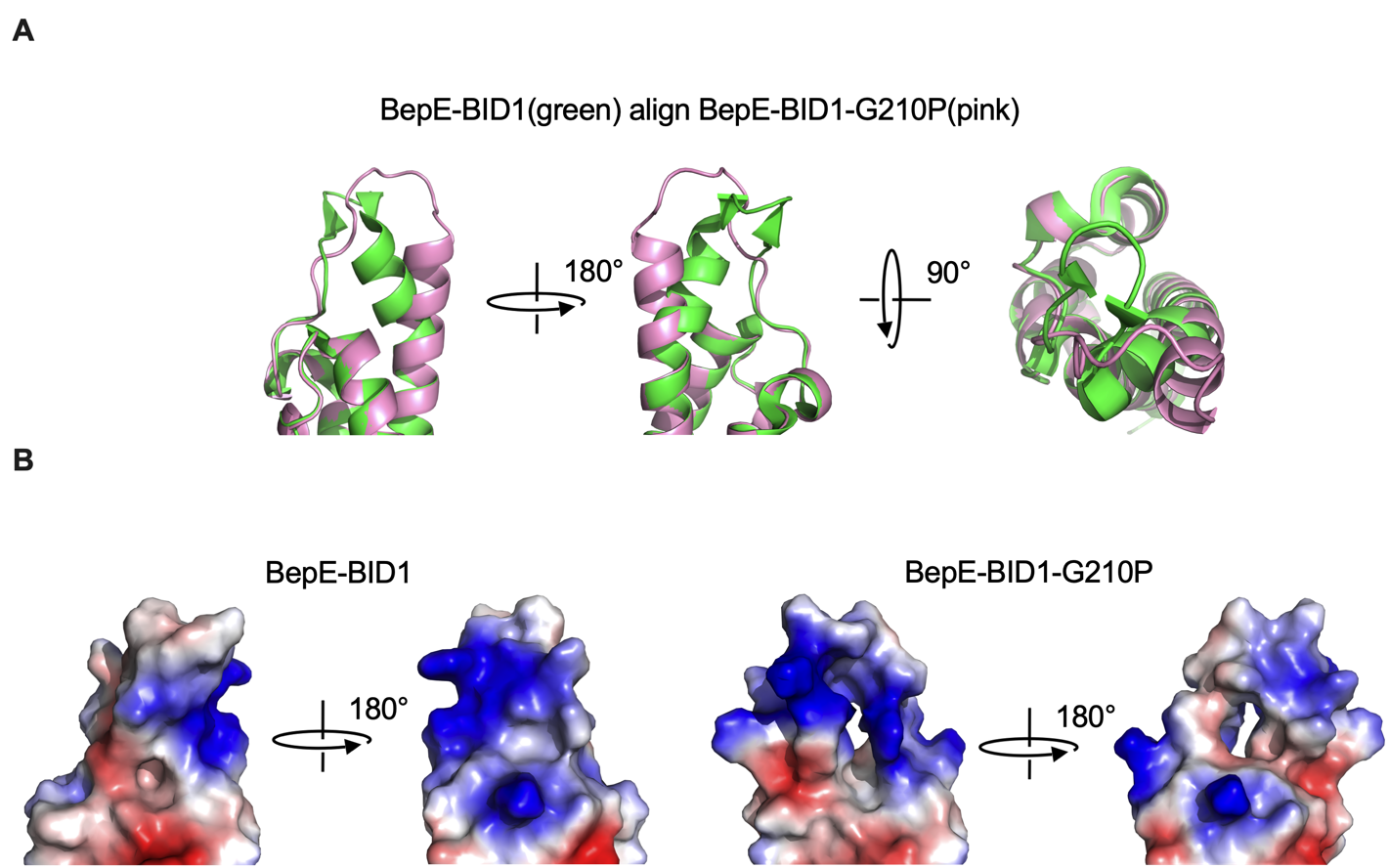

Supplement: S5 Fig — (A) Overlay of Bhe-BepE-BID1 (green, PDB: 4YK3) and Bhe-BepE-BID1-G210P (pink). The predicted structures of Bhe-BepE-BID1-G210P were generated using Alpha-Fold2, and the structural alignment was performed using PyMol. (B) The electrostatic potential of Bhe-BepE-BID1 and Bhe-BepE-BID1-G210P. The protein surfaces are color-coded according to electrostatic potentials, with red representing negative potentials and blue representing positive potentials. (TIF) [file ppat.1012694.s005.tif]
