# Supplementary material for: Anchorage of bacterial effector at plasma membrane via selective phosphatidic acid binding to modulate host cell signaling
Source: PLoS Pathog. 2024 Nov 12;20(11):e1012694. doi: 10.1371/journal.ppat.1012694 (PMC11556746; doi:10.1371/journal.ppat.1012694)
Supplement: S1 Text — (DOCX) [file ppat.1012694.s006.docx]

Supplemental Text 1. The multi-sequence alignments.

(1) The multi-sequence alignments of PA binding motifs from different *Bartonella* species. The figure was generated using ESPript (http://espript.ibcp.fr/ESPript/ESPript)[1].








(2) The multi-sequence alignments of PA binding motifs from different BepD. The figure was generated using ESPript (http://espript.ibcp.fr/ESPript/ESPript)[1].





1. Robert X, Gouet P. Deciphering key features in protein structures with the new ENDscript server. Nucleic Acids Res. 2014;42(Web Server issue):W320-4. Epub 20140421. doi: 10.1093/nar/gku316. PubMed PMID: 24753421; PubMed Central PMCID: PMCPMC4086106.
