# Supplementary material for: Anchorage of bacterial effector at plasma membrane via selective phosphatidic acid binding to modulate host cell signaling
Source: PLoS Pathog. 2024 Nov 12;20(11):e1012694. doi: 10.1371/journal.ppat.1012694 (PMC11556746; doi:10.1371/journal.ppat.1012694)
Supplement: S1 Table — (DOCX) [file ppat.1012694.s008.docx]

Supplemental Table 1. Oligonucleotides used in this study.

| Name | Sequence 5’ - 3’ | Restriction site |
| --- | --- | --- |
| *Bhe*-BepA-eGFP-F | GCTTGGTACCGAGCTCGGATCCATGCCAAAGGCAAAAGCAAAAAC | BamHI |
| *Bhe*-BepA-eGFP-R | CGCCCTTGCTCACCATCTCGAGGCTAGCCATGGCAAGCGTT | XhoI |
| *Bhe*-BepB-eGFP-F | GCTTGGTACCGAGCTCGGATCCATGCCAAAAGCAAAAGCAAAAAATATCTC | BamHI |
| *Bhe*-BepB-eGFP-R | CGCCCTTGCTCACCATCTCGAGGCTGGCAATAGCAAGCGTATTTG | XhoI |
| *Bhe*-BepD-eGFP-F | GCTTGGTACCGAGCTCGGATCCATGAAAAAAAATCGACCATCC | BamHI |
| *Bhe*-BepD-eGFP-R | CGCCCTTGCTCACCATCTCGAGCATACCAAAGGCCATTCCTTTTTG | XhoI |
| *Bhe*-BepF-eGFP-F | GCTTGGTACCGAGCTCGGATCCATGAAAAAAAACCAACCATCC | BamHI |
| *Bhe*-BepF-eGFP-R | CGCCCTTGCTCACCATCTCGAGGAGTGCCAGCACCATTTTTTG | XhoI |
| *Bhe*-BepG-eGFP-F | GCTTGGTACCGAGCTCGGATCCATGAAAAAAAAACAACCAGCCCCTTC | BamHI |
| *Bhe*-BepG-eGFP-R | CGCCCTTGCTCACCATCTCGAGTCTACTCATAGAAACTACTTTTGC | XhoI |
| *Bko*-BepC-eGFP-F | GCTTGGTACCGAGCTCGGATCCATGTTAGAGCAAAATTATCTC | BamHI |
| *Bko*-BepC-eGFP-R | CGCCCTTGCTCACCATCTCGAGGCCGGTAAGAGCCAGCGATGAG | XhoI |
| *Bkr*-BepC-eGFP-F | GCTTGGTACCGAGCTCGGATCCATGTTAGAGAAAAATTATCTCC | BamHI |
| *Bkr*-BepC-eGFP-R | CGCCCTTGCTCACCATCTCGAGGCCGGTAAGAGCTAGCGAAG | XhoI |
| BepC-1-297-F | GCTTGGTACCGAGCTCGGATCCATGTTAGAGCATAATTATCTG | BamHI |
| BepC-1-297-R | CGCCCTTGCTCACCATCTCGAGTATTTCTTTCAGATCGAGAAC | XhoI |
| BepC-BID-F | GCTTGGTACCGAGCTCGGATCCATGTTGATTCCAAAAGAGACCTTG | BamHI |
| BepC-BID-R | CGCCCTTGCTCACCATCTCGAGTCCTGGTTTTTCCACAGAGTG | XhoI |
| BepC-435-532-F | GCTTGGTACCGAGCTCGGATCCATGAGAGATTTGCAACAGCTTTTTAC | BamHI |
| BepC-435-532-R | CGCCCTTGCTCACCATCTCGAGGTTGGTAAGAGCCCTTGCTGAG | XhoI |
| BepA-BID-F | GCTTGGTACCGAGCTCGGATCCATGCTCATCCCAAAAGAGACATTAG | BamHI |
| BepA-BID-R | CGCCCTTGCTCACCATCTCGAGACTTGGCATAGGGACCTCTTG | XhoI |
| BepF-BID1-F | GCTTGGTACCGAGCTCGGATCCATGGCAACAGCTGCTCCATCACAATC | BamHI |
| BepF-BID1-R | CGCCCTTGCTCACCATCTCGAGTTGCGTTCTGTGTGTTTGTTG | XhoI |
| BepE-BID2-F | GCTTGGTACCGAGCTCGGATCCATGCATCCTGAAAGGGAGAGACAATC | BamHI |
| BepE-BID2-R | CGCCCTTGCTCACCATCTCGAGCACTCCAACAGATTGTTCTTG | XhoI |
| *pBepA*-F | CGAGCTCGGTACCCGGGGATCCATGCCAAAGGCAAAAGCAAAAAC | BamHI |
| *pBepA*-R | CGGCTCCACCGCCTCCGTCGACACTTGGCATAGGGACCTCTTGTC | SalI |
| *pBepB*-F | CGAGCTCGGTACCCGGGGATCCATGCCAAAAGCAAAAGCAAAAAATATC | BamHI |
| *pBepB*-R | CGGCTCCACCGCCTCCGTCGACGCTGGCAATAGCAAGCGTATTTG | SalI |
| *pBepC*-F | CGGAGCTCAAGAAGGAGATATACAAATGGACTACAAGGACGACGATGACAAGATGTTAGAGCATAATTATCTG | SacI |
| *pBepC*-R | CGGCGGCCGCCTCAGTTGGTAAGAGCCCTTGCTGAG | NotI |
| *pBepD*-F | CGAGCTCGGTACCCGGGGATCCATGAAAAAAAATCGACCATC | BamHI |
| *pBepD*-R | CGGCTCCACCGCCTCCGTCGACCATACCAAAGGCCATTCCTTTTTG | SalI |
| *pBepE*-F | CGAGCTCGGTACCCGGGGATCCATGAAAAGAAATCAACCACC | BamHI |
| *pBepE*-R | CGGCTCCACCGCCTCCGTCGACGATGGCGAAAGCTATTGCCTTTG | SalI |
| *pBepF*-F | CGAGCTCGGTACCCGGGGATCCATGAAAAAAAACCAACCATCCTC | BamHI |
| *pBepF*-R | CGGCTCCACCGCCTCCGTCGACGAGTGCCAGCACCATTTTTTGTG | SalI |
| *pBepG*-F | CGAGCTCGGTACCCGGGGATCCATGAAAAAAAAACAACCAGCCCCTTC | BamHI |
| *pBepG*-R | CGGCTCCACCGCCTCCGTCGACTCTACTCATAGAAACTACTTTTGC | SalI |
| *pBepD-I-F* | GACGATGACAAGGAGCTCATGAAAAAAAATCGACCATCC | SacI |
| *pBepD-I-R* | GCAGTGCCAGCTTGCGGCCGCTTACATACCAAAGGCCATTCCTTTTTG | NotI |
| *pBepE-I-F* | GACGATGACAAGGAGCTCATGAAAAGAAATCAACCACC | SacI |
| *pBepE-I-R* | GCAGTGCCAGCTTGCGGCCGCTTAGATGGCGAAAGCTATTGCCTTTG | NotI |
| *pBepG-I-F* | GACGATGACAAGGAGCTCATGAAAAAAAAACAACCAGCCCCTTC | SacI |
| *pBepG-I-R* | GCAGTGCCAGCTTGCGGCCGCTTATCTACTCATAGAAACTACTTTTGC | NotI |
| *pBepA-BID*-F | CGAGCTCGGTACCCGGGGATCCATGCTCATCCCAAAAGAGACATTAG | BamHI |
| *pBepA-BID*-R | CGGCTCCACCGCCTCCGTCGACACTTGGCATAGGGACCTCTTG | SalI |
| *pBepA-FIC*-R | CGGCTCCACCGCCTCCGTCGACTTCGGCTTTTGGAGCTGTAAAG | SalI |
| ∆*BepA*-P1-F | ATCCTGACGCCCCCGGGGATCCAGGAGACTTAGAGCGTGACG | BamHI |
| ∆*BepA*-P1-R | GACTTGGCATAGGGACCTCTTGGCGAAGGTATCCAGCCCATTC |  |
| ∆*BepA*-P2-F | GAATGGGCTGGATACCTTCGCCAAGAGGTCCCTATGCCAAGTC |  |
| ∆*BepA*-P2-R | AGCTTAGCTCTGCAGGTCGACGTTCTGAACGGACATACCAATAG | SalI |
| ∆*BepB-G*-P1-F | ATCCTGACGCCCCCGGGGATCCCCACGAGATTATTCAAGAGATG | BamHI |
| ∆*BepB-G*-P1-R | CTTTTTTGTCATTTGCGTTGCCATGTCGCACTGTCCATGAGAG |  |
| ∆*BepB-G*-P2-F | CTCATGGACAGTGCGACATGGCAACGCAAATGACAAAAAAGATC |  |
| ∆*BepB-G*-P2-R | AGCTTAGCTCTGCAGGTCGACGTCTGTCCAGCCCATTCAAAC | SalI |
| ∆*BepG*-P1-F | ATCCTGACGCCCCCGGGGATCCGAGAGGACATTGTAAAAAACC | BamHI |
| ∆*BepG*-P1-R | GTATGCTTATCTACTCATGTGTGTCTCCTTTCGAG |  |
| ∆*BepG-*P2-F | GGAGACACACATGAGTAGATAAGCATACCTGAAAC |  |
| ∆*BepG*-P2-R | AGCTTAGCTCTGCAGGTCGACGAGTCTGTCCAGCCCATTCAAACC | SalI |
| BepE-BID1-His-F | CAGCAAATGGGTCGCGGATCCATGGTTGCTATGCAAAGCACAATACCATC | BamHI |
| BepE-BID1-His-R | GGTGGTGGTGGTGGTGCTCGAGGGCTCTTTGTTCACTCTCTTGTC | XhoI |
| BepD-BID-His-F | CAGCAAATGGGTCGCGGATCCATGCCAAAGGCAAAAGCAAAAAC | BamHI |
| BepD-BID His-R | GGTGGTGGTGGTGGTGCTCGAGTTCGGCTTTTGGAGCTGTAAAG | XhoI |
| BepE-mRFP-F | GACTCAGATCTCGAAGCGCGGCCGCGATGAAAAGAAATCAACCACC | NotI |
| BepE-mRFP-R | GTTATCTAGATCCGGTGGATCCTCAGATGGCGAAAGCTATTGCCTTTG | BamHI |
| PABD-F | GCTTGGTACCGAGCTCGGATCCATGGACAATTGTTCAGGAAGC | BamHI |
| PABD-R | CGCCCTTGCTCACCATCTCGAGCTAAGACTAGTGATGATAAATGC | XhoI |
| BepD-BID-F | CTTGGTACCGAGCTCGGATCCATGCCAGGAAGGGCAACATCTCCTC | BamHI |
| BepD-BID-R | CGCCCTTGCTCACCATCTCGAGCTGCCTTTCTGGGCTTGTCTC | XhoI |
| BepE-K207A-F | CGACGTCTGTTCATGCACTCGCAGGTC |  |
| BepE-K207A-R | GCATGAACAGACGTCGGATTTTCTG |  |
| BepE-R211A-F | CATAAACTCGCAGGTGCCAACCTGTGTG |  |
| BepE-R211A-R | GCACCTGCGAGTTTATGAACAGAC |  |
| BepE-R221A-F | GAAAACAAATGCGGCCAGACAAGCTG |  |
| BepE-R221A-R | GCCGCATTTGTTTTCAAGCCACACAG |  |
| BepE-G210A-F | GTTCATAAACTCGCAGCTCGCAACCTG |  |
| BepE-G210A-R | GCTGCGAGTTTATGAACAGACGTCGG |  |
| BepE-G210P-F | GTTCATAAACTCGCACCTCGCAACCTG |  |
| BepE-G210P-R | GGTGCGAGTTTATGAACAGACGTCGG |  |
| BepE-L213A-F | CTCGCAGGTCGCAACGCGTGTGGCTTG |  |
| BepE-L213A-R | GCGTTGCGACCTGCGAGTTTATGAAC |  |
| BepE-L216A-F | GCAACCTGTGTGGCGCGAAAACAAATG |  |
| BepE-L216A-R | GCGCCACACAGGTTGCGACCTGCGAG |  |
| BepE-L213A-L216A-F | GCAACGCGTGTGGCGCGAAAACAAATG |  |
| BepE-L213A-L216A-R | GCGCCACACGCGTTGCGACCTGCGAG |  |
| BepC-G376P-F | CATTTTGTAAACTTGCGCCGAGGAAAATATTAG |  |
| BepE-G376P-R | GGCGCAAGTTTACAAAATGATTTAG |  |
| BepE-R387A-F | CATAAGAAGTCCAAGTGCCAGGCGTGCCG |  |
| BepE-R387A-R | GCACTTGGACTTCTTATGCCTAATATTTTC |  |
| BepD-D448A-F | GGCATAAAAAGTCCAGCCCGCAAACAAGCTGAAG |  |
| BepD-D448A-R | GCTGGACTTTTTATGCCAAGTATTTTTTGAC |  |
| BepD-D448E-F | GGCATAAAAAGTCCAGAACGCAAACAAGCTGAAG |  |
| BepD-D448E-R | GCTTGTTTGCGTTCTGGACTTTTTATGCCAAG |  |
| *Btr*-BepD-BID-F | GCTTGGTACCGAGCTCGGATCCATGGGCAGAGCAACAACACCTCCTC | BamHI |
| *Btr*-BepD-BID-R | CGCCCTTGCTCACCATCTCGAGACGACGTCTGTTTTCTGGGGTTC | XhoI |
| *Bgr*-BepD-BID-F | GCTTGGTACCGAGCTCGGATCCATGGGCTCAGGCAAGATCTCCCCTC | BamHI |
| *Bgr*-BepD-BID-R | CGCCCTTGCTCACCATCTCGAGGTGTTCTGGGTCTCTTTCTG | XhoI |
| INPP5E-A556D-F | GACGCCCTCATACACGGACCGCGTCTTG |  |
| INPP5E-A556D-R | TCCGTGTATGAGGGCGTCCTCTGCTTG |  |
| FKBP-SAC1-P1-F | GTCCGGACTCAGATCTCGAGGCTCCGGACTCAGATCTCGAAGC | XhoI |
| FKBP-SAC1-P1-R | CCGCCGTCGCCATCGATCGACCTGCTCCTCCTGC |  |
| FKBP-SAC1-P2-F | GAGCAGGTCGATCGATGGCGACGGCGGCCTACGAG |  |
| FKBP-SAC1-P2-R | GTTATCTAGATCCGGTGGATCCTCAGTCTATCTTTTCTTTCTGGAC | BamHI |
| Lyn_11_-FRB-BFP-F | CTACCGGACTCAGATCTCGAGATGGGATGTATAAAATCAAAAG | XhoI |
| Lyn_11_-FRB-BFP-R | CATGGTGGCGACCGGTGGATCCCCTGCGTAGTCTGGTACGTCGTAC | BamHI |
