# Supplementary material for: Anchorage of bacterial effector at plasma membrane via selective phosphatidic acid binding to modulate host cell signaling
Source: PLoS Pathog. 2024 Nov 12;20(11):e1012694. doi: 10.1371/journal.ppat.1012694 (PMC11556746; doi:10.1371/journal.ppat.1012694)
Supplement: S2 Table — (DOCX) [file ppat.1012694.s009.docx]

Supplemental Table 2. List and construction of all plasmids used in this study.

| Plasmid name | Description | vector backbone | Primers | Template for PCR | Source/ Reference |
| --- | --- | --- | --- | --- | --- |
| pcDNA4.0-eGFP | Mammalian expression vector with eGFP tag | pcDNA™4/TO |  |  | (1) |
| eGFP-*Bhe-*BepA | Mammalian expression vector for *Bhe-*BepA with eGFP tag | pcDNA™4/TO | *Bhe*-BepA-eGFP-F/  *Bhe*-BepA-eGFP-R | boiled colony of *Bhe-*JK33 | This study |
| eGFP-*Bhe-*BepB | Mammalian expression vector for *Bhe-*BepB with eGFP tag | pcDNA™4/TO | *Bhe*-BepB-eGFP-F/  *Bhe*-BepB-eGFP-R | boiled colony of *Bhe-*JK33 | This study |
| eGFP-*Bhe-*BepC | Mammalian expression vector for *Bhe-*BepC with eGFP tag | pcDNA™4/TO |  |  | (1) |
| eGFP-*Bhe-*BepD | Mammalian expression vector for *Bhe-*BepD with eGFP tag | pcDNA™4/TO | *Bhe*-BepD-eGFP-F/  *Bhe*-BepD-eGFP-R | boiled colony of *Bhe-*JK33 | This study |
| eGFP-*Bhe-*BepE | Mammalian expression vector for *Bhe-*BepE with eGFP tag | pcDNA™4/TO | *Bhe*-BepE-eGFP-F/  *Bhe*-BepE-eGFP-R | boiled colony of *Bhe-*JK33 | (2) |
| eGFP-*Bhe-*BepF | Mammalian expression vector for *Bhe-*BepF with eGFP tag | pcDNA™4/TO | *Bhe*-BepF-eGFP-F/  *Bhe*-BepF-eGFP-R | boiled colony of *Bhe-*JK33 | This study |
| eGFP-*Bhe-*BepG | Mammalian expression vector for *Bhe-*BepG with eGFP tag | pcDNA™4/TO | *Bhe*-BepG-eGFP-F/  *Bhe*-BepG-eGFP-R | boiled colony of *Bhe-*JK33 | This study |
| eGFP-*Btr*-BepC | Mammalian expression vector for *Btr-*BepC with eGFP tag | pcDNA™4/TO |  |  | (1) |
| eGFP-*Bko-*BepC | Mammalian expression vector for *Bko-*BepC with eGFP tag | pcDNA™4/TO | *Bko-*BepC-eGFP-F/  *Bko-*BepC-eGFP-R | boiled colony of *Bko* Strain *Tel Aviv* | This study |
| eGFP-*Bkr-*BepC | Mammalian expression vector for *Bkr-*BepC with eGFP tag | pcDNA™4/TO | *Bkr-*BepC-eGFP-F/  *Bkr-*BepC-eGFP-R | boiled colony of *Bkr* Strain *OE 1-1* | This study |
| eGFP-*Bqu-*BepC | Mammalian expression vector for *Bqu-*BepC with eGFP tag | pcDNA™4/TO |  |  | (1) |
| eGFP-BepC-(1-297) | Mammalian expression vector for *Bhe-*BepC-(1-297) with eGFP tag | pcDNA™4/TO | BepC-1-297-F/  BepC-1-297-R | boiled colony of *Bhe-*JK33 | This study |
| eGFP-BepC-BID | Mammalian expression vector for *Bhe-*BepC-BID with eGFP tag | pcDNA™4/TO | BepC-BID-F/  BepC-BID-R | boiled colony of *Bhe-*JK33 | This study |
| eGFP-BepC-(435-532) | Mammalian expression vector for *Bhe-*BepC-(435-532) with eGFP tag | pcDNA™4/TO | BepC-435-532-F/  BepC-435-532-R | boiled colony of *Bhe-*JK33 | This study |
| eGFP-BepA-BID | Mammalian expression vector for *Bhe-*BepA-BID with eGFP tag | pcDNA™4/TO | BepA-BID-F/  BepA-BID-R | boiled colony of *Bhe-*JK33 | This study |
| eGFP-BepF-BID1 | Mammalian expression vector for *Bhe-*BepF-BID1 with eGFP tag | pcDNA™4/TO | BepF-BID1-F/  BepF-BID1-R | boiled colony of *Bhe-*JK33 | This study |
| eGFP-BepE-BID2 | Mammalian expression vector for *Bhe-*BepE-BID2 with eGFP tag | pcDNA™4/TO | BepE-BID2-F/  BepE-BID2-R | boiled colony of *Bhe-*JK33 | This study |
| *pANT4-Flag* | Prokaryotic expression vector | pANT4 (3) |  | *3 x Flag tag* was *synthesized by TSINGKE Biological Technology* | This study |
| *pBepA* | Prokaryotic expression vector for *Bhe*-BepA with FLAG tag | pANT4 | *pBepA*-F/  *pBepA*-R | boiled colony of *Bhe-*JK33 | This study |
| *pBepB* | Prokaryotic expression vector for *Bhe*-BepB with FLAG tag | pANT4 | *pBepB*-F/  *pBepB*-R | boiled colony of *Bhe-*JK33 | This study |
| *pBepC* | IPTG-induced prokaryotic expression vector for *Bhe*-BepC with FLAG tag | *pBZ485* |  |  | (4) |
| *pBepD* | Prokaryotic expression vector for *Bhe*-BepD with FLAG tag | pANT4 | *pBepD*-F/  *pBepD*-R | boiled colony of *Bhe-*JK33 | This study |
| *pBepE* | Prokaryotic expression vector for *Bhe*-BepE with FLAG tag | pANT4 | *pBepE*-F/  *pBepE*-R | boiled colony of *Bhe-*JK33 | This study |
| *pBepF* | Prokaryotic expression vector for *Bhe*-BepF with FLAG tag | pANT4 | *pBepF*-F/  *pBepF*-R | boiled colony of *Bhe-*JK33 | This study |
| *pBepG* | Prokaryotic expression vector for *Bhe*-BepG with FLAG tag | pANT4 | *pBepG*-F/  *pBepG-*R | boiled colony of *Bhe-*JK33 | This study |
| *pBepD-I* | IPTG-induced prokaryotic expression vector for *Bhe*-BepD with FLAG tag | *pBZ485* | *pBepD-I*-F/  *pBepD-I*-R | boiled colony of *Bhe-*JK33 | This study |
| *pBepD-D448A-I* | IPTG-induced prokaryotic expression vector for *Bhe*-BepD-D448A with FLAG tag | *pBZ485* | BepD-D448A-F/  BepD-D448A-R | pBepD-I | This study |
| *pBepE-I* | IPTG-induced prokaryotic expression vector for *Bhe*-BepE with FLAG tag | *pBZ485* | *pBepE-I*-F/  *pBepE-I*-R | boiled colony of *Bhe-*JK33 | This study |
| *pBepG-I* | IPTG-induced prokaryotic expression vector for *Bhe*-BepG with FLAG tag | *pBZ485* | *pBepG-I*-F/  *pBepG-I*-R | boiled colony of *Bhe-*JK33 | This study |
| *pBepA-BID* | Prokaryotic expression vector for *Bhe*-BepA-BID with FLAG tag | pANT4 | *pBepA-BID-*F/  *pBepA-BID*-R | boiled colony of *Bhe-*JK33 | This study |
| *pBepA-FIC* | Prokaryotic expression vector for *Bhe*-BepA-FIC with FLAG tag | pANT4 | *pBepA* -F/  *pBepA-FIC*-R | boiled colony of *Bhe-*JK33 | This study |
| ∆*bepA* | Deletion of BepA in WT background | pJM05 | ∆BepA-P1-F/R  ∆BepA-P2-F/R | boiled colony of *Bhe-*JK33 | This study |
| ∆*bepB-G* | Deletion of BepB-G in ∆BepA | pJM05 | ∆BepG-P1-F/R  ∆BepG-P2-F/R | boiled colony of *Bhe-*JK33 | This study |
| ∆*bepG* | Deletion of BepG in WT background | pJM05 | ∆BepB-G-P1-F/R  ∆BepB-G-P2-F/R | boiled colony of *Bhe-*JK33 | This study |
| BepE-BID1-His | Prokaryotic expression vector for *Bhe*-BepE-BID1 with His tag | pET-28a(+) | BepE-BID1-His-F/  BepE-BID1-His-F | boiled colony of *Bhe-*JK33 | This study |
| BepD-BID-His | Prokaryotic expression vector for *Bhe*-BepD-BID with His tag | pET-28a(+) | BepD-BID-His-F/  BepD-BID His-R | eGFP-BepD-BID | This study |
| BepD-BID-D448A-His | Prokaryotic expression vector for *Bhe*-BepD-BID-D448A with His tag | pET-28a(+) | BepD-BID-His-F/  BepD-BID His-R | eGFP-BepD-BID-D448A | This study |
| BepD-BID-D448E-His | Prokaryotic expression vector for *Bhe*-BepE-BID-D448E with His tag | pET-28a(+) | BepD-BID-His-F/  BepD-BID His-R | eGFP-BepD-BID-D448E | This study |
| PABD-GFP | Mammalian expression vector for spo20p (51-91) with eGFP tag | pcDNA™4/TO | PABD-F/  PABD-R | *cDNA* library from Saccharomyces cerevisiae | This study |
| P4M-GFP | Mammalian expression vector for *SidM(546-647)* with eGFP tag | pcDNA™4/TO |  | *L. pneumophila SidM(546-647)* was *synthesized by TSINGKE Biological Technology* | This study |
| PH-PLCD1-GFP | Mammalian expression vector for PH-PLCD1 (1–170) with eGFP tag | pEGFP-N1 |  |  | (5) Addgene plasmid # 51407 |
| PH-Btk-GFP | Mammalian expression vector for BTK (1–177) with eGFP tag | pEGFP-N1 |  |  | (5) Addgene plasmid # 51463 |
| mRFP-BepE | Mammalian expression vector for *Bhe*-BepE with mRFP tag | mRFP-C1 | BepE-mRFP-F/  BepE-mRFP-R | boiled colony of *Bhe-*JK33 | This study |
| eGFP-BepE-BID1-K207A | Mammalian expression vector for *Bhe-*BepE-BID1-K207A with eGFP tag | pcDNA™4/TO | BepE-K207A-F/  BepE-K207A-R | eGFP-BepE-BID1 | This study |
| eGFP-BepE-BID1-R211A | Mammalian expression vector for *Bhe-*BepE-BID1-R211A with eGFP tag | pcDNA™4/TO | BepE-R211A-F/  BepE-R211A-R | eGFP-BepE-BID1 | This study |
| eGFP-BepE-BID1-R221A | Mammalian expression vector for *Bhe-*BepE-BID1- R221A with eGFP tag | pcDNA™4/TO | BepE-R221A-F/  BepE-R221A-R | eGFP-BepE-BID1 | This study |
| eGFP-BepE-BID1-G210A | Mammalian expression vector for *Bhe-*BepE-BID1- G210A with eGFP tag | pcDNA™4/TO | BepE-G210A-F/  BepE-G210A-R | eGFP-BepE-BID1 | This study |
| eGFP-BepE-BID1-G210P | Mammalian expression vector for *Bhe-*BepE-BID1- G210P with eGFP tag | pcDNA™4/TO | BepE-G210P -F/  BepE-G210P -R | eGFP-BepE-BID1 | This study |
| eGFP-BepE-BID1-L213A | Mammalian expression vector for *Bhe-*BepE-BID1- L213A with eGFP tag | pcDNA™4/TO | BepE-L213A -F/  BepE-L213A -R | eGFP-BepE-BID1 | This study |
| eGFP-BepE-BID1-L216A | Mammalian expression vector for *Bhe-*BepE-BID1- L216A with eGFP tag | pcDNA™4/TO | BepE-L216A -F/  BepE-L216A -R | eGFP-BepE-BID1 | This study |
| eGFP-BepE-BID1-L213A-L216A | Mammalian expression vector for *Bhe-*BepE-BID1- L213A-L216A with eGFP tag | pcDNA™4/TO | BepE-L213A-L216A -F/  BepE-L213A-L216A -R | eGFP-BepE-BID1 | This study |
| mRFP-FKBP-INPP5E | Mammalian expression vector for FKBP1A (3-108) + INPP5E with mRFP tag | mRFP-C1 | INPP5E-A556D-F/  INPP5E-A556D-R | mRFP-FKBP-INPP5E(D556A)  (6) Addgene plasmid # 183678 | This study |
| mCherry-FKBP-SAC1 | Mammalian expression vector for FKBP1A (3-108) + SACM1L with mcherry tag | mCherry-C1 | FKBP-SAC1-P1-F/R  FKBP-SAC1-P2-F/R | *cDNA* library from HeLa cells/  mRFP-FKBP-INPP5E | This study |
| LYN_11_-FRB-BFP | Mammalian expression vector for LYN (1-11)  + MTOR (2021–2113)  with mTagBFP2 tag | pmTagBFP2-N | Lyn_11_-FRB-BFP-F/  Lyn_11_-FRB-BFP-R | LYN11-FRB-mcherry (7) Addgene plasmid # 38004 | This study |
| eGFP-BepC-G376P | Mammalian expression vector for *Bhe-*BepC-G376P with eGFP tag | pcDNA™4/TO | BepC-G376P-F/  BepC-G376P-R | eGFP-*Bhe-*BepC | This study |
| eGFP-BepC-R387A | Mammalian expression vector for *Bhe-*BepC-R387A with eGFP tag | pcDNA™4/TO | BepC-R387A-F/  BepC-R387A-R | eGFP-*Bhe-*BepC | This study |
| eGFP-BepC-G376P-R387A | Mammalian expression vector for *Bhe-*BepC-G376P-R387A with eGFP tag | pcDNA™4/TO | BepC-R387A-F/  BepC-R387A-R | eGFP-BepC-G376P | This study |
| eGFP-BepD-BID | Mammalian expression vector for *Bhe-*BepD-BID with eGFP tag | pcDNA™4/TO | BepD-BID-F/  BepD-BID-R | eGFP-*Bhe-*BepD | This study |
| eGFP-BepD-BID-D448A | Mammalian expression vector for *Bhe-*BepD-BID-D448A with eGFP tag | pcDNA™4/TO | BepD-D448A-F/  BepD-D448A-R | eGFP-BepD-BID | This study |
| eGFP-BepD-BID-D448E | Mammalian expression vector for *Bhe-*BepD-BID- D448E with eGFP tag | pcDNA™4/TO | BepD-D448E-F/  BepD-D448E-R | eGFP-BepD-BID | This study |
| eGFP-*Btr*-BepD-BID | Mammalian expression vector for *Btr*-BepD-BID with eGFP tag | pcDNA™4/TO | *Btr*-BepD-BID-F/  *Btr*-BepD-BID-R | boiled colony of *Btr* | This study |
| eGFP-*Bgr*-BepD-BID | Mammalian expression vector for *Bgr*-BepD-BID with eGFP tag | pcDNA™4/TO | *Bgr*-BepD-BID-F/  *Bgr*-BepD-BID-R | boiled colony of *Bgr* | This study |

**SI References**

1. C. Wang *et al.*, Bartonella type IV secretion effector BepC induces stress fiber formation through activation of GEF-H1. *PLoS Pathog* **17**, e1009065 (2021).

2. C. Wang *et al.*, Bartonella quintana type IV secretion effector BepE-induced selective autophagy by conjugation with K63 polyubiquitin chain. *Cell Microbiol* **21**, e12984 (2019).

3. A. K. Lee, S. Falkow, Constitutive and inducible green fluorescent protein expression in Bartonella henselae. *Infect Immun* **66**, 3964-3967 (1998).

4. S. Marlaire, C. Dehio, Bartonella effector protein C mediates actin stress fiber formation via recruitment of GEF-H1 to the plasma membrane. *PLoS Pathog* **17**, e1008548 (2021).

5. P. Várnai, T. Balla, Visualization of phosphoinositides that bind pleckstrin homology domains: calcium- and agonist-induced dynamic changes and relationship to myo-[3H]inositol-labeled phosphoinositide pools. *J Cell Biol* **143**, 501-510 (1998).

6. G. F. W. Walpole *et al.*, Kinase-independent synthesis of 3-phosphorylated phosphoinositides by a phosphotransferase. *Nat Cell Biol* **24**, 708-722 (2022).

7. G. R. Hammond *et al.*, PI4P and PI(4,5)P2 are essential but independent lipid determinants of membrane identity. *Science* **337**, 727-730 (2012).
