# Supplementary material for: Anchorage of bacterial effector at plasma membrane via selective phosphatidic acid binding to modulate host cell signaling
Source: PLoS Pathog. 2024 Nov 12;20(11):e1012694. doi: 10.1371/journal.ppat.1012694 (PMC11556746; doi:10.1371/journal.ppat.1012694)
Supplement: S3 Table — (DOCX) [file ppat.1012694.s010.docx]

Supplemental Table 3. Bacterial Strains.

| Name | Source |
| --- | --- |
| *Bartonella henselae strain* *JK33* | [1] |
| *JK33-∆bepA-G* | This study |
| *JK33-∆bepA-G-pBepA* | This study |
| *JK33-∆bepA-G-pBepB* | This study |
| *pBepC* | [2] |
| *JK33-∆bepA-G-pBepD* | This study |
| *JK33-∆bepA-G-pBepE* | This study |
| *JK33-∆bepA-G-pBepF* | This study |
| *JK33-∆bepA-G-pBepG* | This study |
| *JK33-∆bepA-G-pBepA-BID* | This study |
| *JK33-∆bepA-G-pBepA-FIC* | This study |
| *JK33-∆bepA-G-pFlag* | This study |
| *JK33-∆bepA-G-pBepD-D448A* | This study |
| *JK33-∆bepG* | This study |
| *JK33-∆bepG-pBepG* | This study |
| *Bartonella quintana strain* *JK31* | [2] |
| *Bartonella tribocorrum* | This study |
| *Bartonella kosoyi strain Tal aviv* | [3] |
| *Bartonella krasnovii strain OE 1-1* | [3] |
| *E. coli, DH5a* | Weidi Biotechnology (Cat# DL1001) |
| *E. coli, BL21(DE3)* | Weidi Biotechnology (Cat# EC1002) |
| *E. coli, S17-1 λpir* | Weidi Biotechnology (Cat# DL2010) |

1. Rodriguez-Barradas MC, Hamill RJ, Houston ED, Georghiou PR, Clarridge JE, Regnery RL, et al. Genomic fingerprinting of Bartonella species by repetitive element PCR for distinguishing species and isolates. J Clin Microbiol. 1995;33(5):1089-93. Epub 1995/05/01. doi: 10.1128/jcm.33.5.1089-1093.1995. PubMed PMID: 7615711; PubMed Central PMCID: PMCPMC228110.

2. Wang C, Zhang H, Fu J, Wang M, Cai Y, Ding T, et al. Bartonella type IV secretion effector BepC induces stress fiber formation through activation of GEF-H1. PLoS Pathog. 2021;17(1):e1009065. Epub 2021/01/29. doi: 10.1371/journal.ppat.1009065. PubMed PMID: 33508039; PubMed Central PMCID: PMCPMC7842913.

3. Gutiérrez R, Shalit T, Markus B, Yuan C, Nachum-Biala Y, Elad D, et al. Bartonella kosoyi sp. nov. and Bartonella krasnovii sp. nov., two novel species closely related to the zoonotic Bartonella elizabethae, isolated from black rats and wild desert rodent-fleas. Int J Syst Evol Microbiol. 2020;70(3):1656-65. doi: 10.1099/ijsem.0.003952. PubMed PMID: 32100689.
